# Systematic Review and Meta-Analysis of the Impact of Carer Stress on Subsequent Institutionalisation of Community-Dwelling Older People

**DOI:** 10.1371/journal.pone.0128213

**Published:** 2015-06-02

**Authors:** Nora-Ann Donnelly, Anne Hickey, Annette Burns, Paul Murphy, Frank Doyle

**Affiliations:** 1 Department of Psychology, Royal College of Surgeons in Ireland, Dublin, Ireland; 2 Mercer Library, Royal College of Surgeons in Ireland, Dublin, Ireland; University of Louisville, UNITED STATES

## Abstract

**Background:**

In the caregiving literature there is a common assertion that a higher level of carer stress is a critical determinant of premature ending of homecare. However, this contention has not been systematically assessed. We therefore systematically reviewed and meta-analysed the prospective association between various forms of carer stress and subsequent institutionalisation of community-dwelling older people.

**Methods:**

Systematic literature search of prospective studies measuring carer stress at baseline and institutionalisation at follow-up. Given substantial interchangeability in the measurement of carer stress, we included a wide number of exposure measures, namely: carer stress, burden, depression, distress, anxiety, burnout, and strain. Institutionalisation included both acute and long-term care utilisation. The standardised mean difference between stressed and non-stressed carers was the primary measure of effect. We assessed study quality with the Crowe Critical Appraisal Tool (CCAT). Pre-planned sensitivity analysis included examination of estimates according to study size; decade published; study quality according to quartiles of CCAT scores; population; follow-up period; study design and impact of adjusted or unadjusted estimates.

**Results:**

The search yielded 6,963 articles. After exclusions, we analysed data from 54 datasets. The meta-analysis found that while carer stress has a significant effect on subsequent institutionalisation of care recipients, the overall effect size was negligible (SMD=0·05, 95% CI=0·04–0·07). Sensitivity analyses found that, the effect size was higher for measurements of stress than for other measures, though still relatively small (SMD=0·23, 95% CI=0·09–0·38). Thus, whether analysing the association between carer stress, burden, distress, or depression with either acute or long-term care, the effect size remains small to negligible. Concurrently, we found estimates reduce over time and were smaller with larger studies and those of higher quality, according to the CCAT scores.

**Conclusion:**

Despite strong statements to the contrary, it appears that the effect of carer stress on subsequent care recipient institutionalisation is small to negligible. The current findings point to a biased literature, with significant small study effects. The results suggest a need to re-evaluate the degree to which carer stress predicts premature ending of home care. Concurrently, other factors may be more crucial in institutional placement than carer stress and should be investigated.

## Introduction

The caregiving role provided by family members and informal carers to older care recipients is often regarded as hazardous to a carer’s psychological well-being and physical health. Indeed, a number of meta-analyses and systematic reviews have concluded that carers are more likely to have poor psychological and physical health outcomes when compared to non-carers [1, 2]. Therefore, in examining the sustainability of home care, gerontological researchers have increasingly recognised the needs of the carer as well as the care recipient. This work has given much attention to the psychological health effects of caregiving, leading to a contention that such psychological morbidity of the carer could increase the risk of institutionalisation of the care recipient.

Before reviewing this contention it is important to note that, while there is extensive literature on the psychological health effects of caregiving, particularly carer stress, burden, and depression, there is a lack of clarity on their distinction and how they interact in the caregiving process. Indeed, though burden is one of the most commonly analysed variables in the literature, there is no single definition or uniform conceptualisation of carer burden [3, 4]. A number of researchers have acknowledged this lack of clarity by framing the examination of carer burden within the context of a stress process model [5–7]. Critical to the understanding of burden is the model’s distinction between objective and subjective primary stressors [8]. Objective stressors include care recipient’s functional disability and behaviours that challenge. Subjective stressors are the carer’s appraisal of objective stressors. Thus, within this model, burden is not understood as a separate construct from stress, rather it is a carer’s subjective appraisal of his or her situation [6, 8, 9]. We have adopted this interpretation of carer burden and stress for the purposes of this systematic review.

Despite attempts to provide clarity around the psychological health effects of caregiving, virtually every dimension of the stress process (from the primary stressors such as behaviours that challenge, to the outcomes of the model, including anxiety and depression) has been referred to as burden [10]. Consequently, the term ‘burden’ has been used in many different ways in the literature. Furthermore, there is a tendency of some researchers to use the concepts and associated measures interchangeably [4]. For example, studies discuss ‘carer stress’ yet apply measures of burden [11], measures of depression [12], or measures of strain [13]; or assess ‘carer strain’ and apply measures of distress [14]; or refer to ‘carer burden’ as synonymous with carer burnout [15]. Given the lack of clarity and consequential interchangeability in the measurement of psychological morbidity, any review of the area must incorporate a wide number of exposures employed to measure carer psychological morbidity. Brodaty et al., also adopted this approach in a similar systematic review [16]. Therefore, in order to avoid omitting a substantial proportion of the literature, we had to include a wide number of exposures that are measured under the umbrella term of ‘carer stress’, namely: stress, burden, depression, distress, anxiety, burnout, and strain.

As referred to above, in the caregiving literature there is a commonly held assertion that, as a carer is a critical element of home care, if the level of stress on a carer becomes too great, the home care support provided by the carer may be jeopardized [17]. Indeed, a number of cohort studies have found that higher levels of caregiver stress, burden, and depression can predict admission of the care recipient into a nursing home [13, 18, 19]. Others authors suggest that carer stress was the principal determinant of nursing home placement [20]. Kuzuya et al., also found carer burden to be an important risk factor for hospitalisation of care recipients [21]. This same study suggests that interventions aiming to reduce carer burden and improve carer well-being could delay long-term placement [21]. Furthermore, a recent publication in the Lancet Psychiatry [22] stated that “carer psychological morbidity predicts care breakdown and care home admissions” (p. 1). However, the assertion that higher levels of carer stress could jeopardize home care has not been subject to meta-analysis.

Indeed, the majority of reviews of the predictors of institutionalisation have not accounted for the level of carer stress [23–27]. Gaugler et al., did include carer stress in a systematic review of factors that consistently predict nursing home admission in people with dementia [28]. This review found that, carers who indicated greater emotional stress were more likely to admit the care recipient to a nursing home. While this review considered separate measures of carer stress, the analysis was confined to whether carer stress was a significant predictor and the direction of the effect (positive or negative). However, unlike the current study, the review did not analyse the size of the effect [28]. Finally, when both carer and care recipient characteristics have been analysed together in cohort studies, the strength of the association between carer stress and institutionalisation has varied between studies, suggesting potential heterogeneity in these effects [18, 29, 30].

Given the absence of a systematic review and meta-analysis in the area, we systematically reviewed and meta-analysed the prospective association between carer stress and institutional placement of the care recipient. For the purposes of the review, ‘carer stress’ is used as an umbrella term to incorporate the wide number of exposures that are used synonymously in the measurement of the psychological health effects of caregiving.

### Objective

To examine the effect of carer stress on subsequent institutional placement of community-dwelling older people.

## Methods

### Study design

The PRISMA guidelines for the conduct and reporting of systematic reviews and meta-analyses were adhered to in the conduct of this review [31].

### Eligibility criteria

#### Types of studies

Both naturalistic observational and intervention studies that measured carer stress at baseline and acute or long-term care utilisation at follow-up were included. We assessed prospective observational studies; control groups from controlled intervention studies with carers and, where data from control groups alone could not be obtained, combined intervention and control groups were also included. Sensitivity analyses examined the differential effect of study design on estimates and the effect of excluding those studies where data from control groups alone could not be obtained. We excluded studies if they were cross-sectional, retrospective or not written in English. Articles were not limited by year of publication.

#### Types of participants

Care recipients: Community-dwelling older people (aged 65 and over) with chronic care needs that are being cared for by an informal carer. We did not confine the study to participants of a particular demographic group or ethnicity. Thus participants with Dementia or other chronic disabilities who have an established caregiving arrangement in the community were included. Carer: Informal carer who takes primary responsibility of the care recipient. We excluded articles with data on professional or paid carers.

#### Types of exposures

As mentioned above, different measures of psychological morbidity have been analysed in the prediction of institutional placement. In order to avoid omitting a substantial proportion of the literature, we had to include a wide number of exposure measures that are used under the umbrella term ‘carer stress’, namely: stress, burden, depression, distress, anxiety, burnout, and strain. Brodaty et al., also employed this approach in a similar systematic review [16]. Given the range of possible exposure variables, we considered them in a hierarchical manner, with composite measures of burden and stress which have been tested for validity and reliability given priority. These were followed by composite measures of depression, distress, anxiety, or strain which have also been tested for validity and reliability. Where studies included more than one measure, we recorded both and analysed these separately in a sensitivity analysis. This enabled an examination of differences between estimates solely with measures of stress, burden, depression or distress. It was also possible to examine estimates solely with measures of burden and stress according to the stress process model [6] and with measures of psychological distress, as adopted in a similar systematic review [16]. For the overall effect estimate we employed the above hierarchy to select the best estimate.

#### Types of outcomes

Acute care utilisation: Emergency Department visits and/or hospital admissions. Long-term care utilisation: Admission to a nursing home.

### Search methods

#### Information sources

We undertook a systematic literature search in January 2014 in the following databases: CINAHL, Medline (OVID), PsycInfo, Web of Knowledge, and EMBASE.

#### Search terms

The search terms were: carer or caregiver; aged or elderly or Alzheimer or dementia; stress or burden or burnout or distress or anxiety or depression or strain; nursing home or long term care or long term care utilisation or care home or homes for the aged or institutionalisation or acute care or hospitalisation or hospital admission or hospital readmission or emergency department or accident and emergency. S1 Appendix provides an example of the search strategy for Medline (OVID).

### Data collection and analysis

#### Study selection

The first reviewer screened all titles and abstracts of papers identified by the literature search (NAD). Given resource constraints, a second reviewer (AB) undertook duplicate screening on a random selection of fifteen percent of found titles/abstracts. We discussed disagreements with a third reviewer (FD). All studies identified as potentially relevant were retrieved and read in full to determine eligibility for inclusion.

#### Data extraction

We conducted data extraction by using a pre-defined data extraction template. Extracted data included design characteristics; study population and country; sample size; length of follow up; sample selection; age and sex of participants; the exposure and outcome measures; and results. Where there were insufficient data in the published paper we contacted authors to provide further information.

#### Quality assessment

We conducted quality assessment with the Crowe Critical Appraisal Tool (CCAT) [32–34]. The CCAT was developed based on a wide number of previous critical appraisal tools, general research methods theory and reporting guidelines [32]. The tool has undergone testing for reliability and validity, with Crowe et al., reporting intraclass correlation coefficients of 0.83 (consistency) and 0.74 (absolute agreement) [32, 33]. A similar recent meta-analysis also employed the CCAT to assess study quality [35].

Based on the CCAT, we appraised papers included in the review in eight categories. These were preliminary appraisals (such as the title and abstract), the introduction, design, sampling, data collection, ethical matters, results, and discussion. Within each category we examined a number of items such as sampling method, sample size and bias. Scoring was a combination of objective and subjective assessment, where each category is scored from 0 (no evidence) to 5 (highest evidence). Total scores for each study are presented as a percentage. Thus the tool enables direct comparison of scores obtained in the quality assessment of articles included in the review [32, 34]. In the sensitivity analysis, to examine the impact of study quality on effect estimates in a meaningful way, we grouped studies by quartiles of CCAT scores. However, we maintained continuous scores for the meta-regression.

#### Statistical analysis

The standardised mean difference (SMD) between stressed and non-stressed carers was the primary measure of effect. This approach is recommended when there is variance in measurement of exposures (e.g., mean and SD of stress, burden, or depression scores or proportions stressed, burdened, or depressed) and outcome status (acute or long term care utilisation) [36, 37]. As studies reported a combination of mean and SD scores or proportions stressed or not, we employed the metaeff command in Stata 12.0 [38]. This command enabled the calculation an effect size and its standard error by using methods described in the Cochrane Handbook of Systematic Reviews of Interventions [38, 39]. Thus data were transformed to a common effect size metric. We estimated effects in a random effects model [37] for all included studies. We employed the I^2^ test to describe the percentage of total variation across studies that was due to heterogeneity rather than chance [40]. We conducted an assessment of publication bias or small study effects visually with a funnel plot and more formally with Egger’s test.

We conducted a pre-planned sensitivity analysis of estimates according to study size by tertile; the decade studies were published and regions in which studies were conducted; study quality by quartiles of CCAT scores; use of adjusted or unadjusted estimates; dementia populations compared to non-dementia populations; different follow-up periods; study design and long-term care in comparison to acute care utilisation. We examined the impact of different exposure measures in a number of ways. Firstly, we examined differences between estimates solely with measures of stress, burden, depression or distress separately. Where the same scale was used to measure ‘stress’, ‘distress’ and ‘burden’, we applied the original classification by the authors of the scale. For example, the General Health Questionnaire was classified as ‘distress’, an approach also adopted in a similar systematic review [41, 42]. We compared differences in estimates between studies applying the Zarit Burden Interview (ZBI) with studies that applied other measures of burden. It was also possible to examine estimates solely with measures of burden and stress, according to the stress process model [6], and with measures of psychological distress, as adopted in a similar systematic review [16]. We also examined differences in estimates within studies that measured both burden and depression. Finally, we did a further sensitivity analysis of those studies that applied measures of stress. Here, we examined the effect estimates for studies which used a validated measure of stress (that is the psychometric properties of the scale have been reported), in comparison with those studies that did not use a validated measure or report if the psychometric properties of the scale had been tested.

Given the methodological diversity of included studies, we anticipated significant heterogeneity. Therefore we planned a meta-regression to understand the extent to which heterogeneity was related to the characteristics of the studies [43]. This included the year studies were published, study size and quality, whether the estimates were adjusted for, the type of outcome and exposure measure and the period of follow-up. We examined each study characteristic individually first. We then entered study characteristics found to be significant into a meta-regression with multiple covariates to assess their overall contribution to heterogeneity in effect estimates [43].

## Results

### Study selection

**Fig 1:**
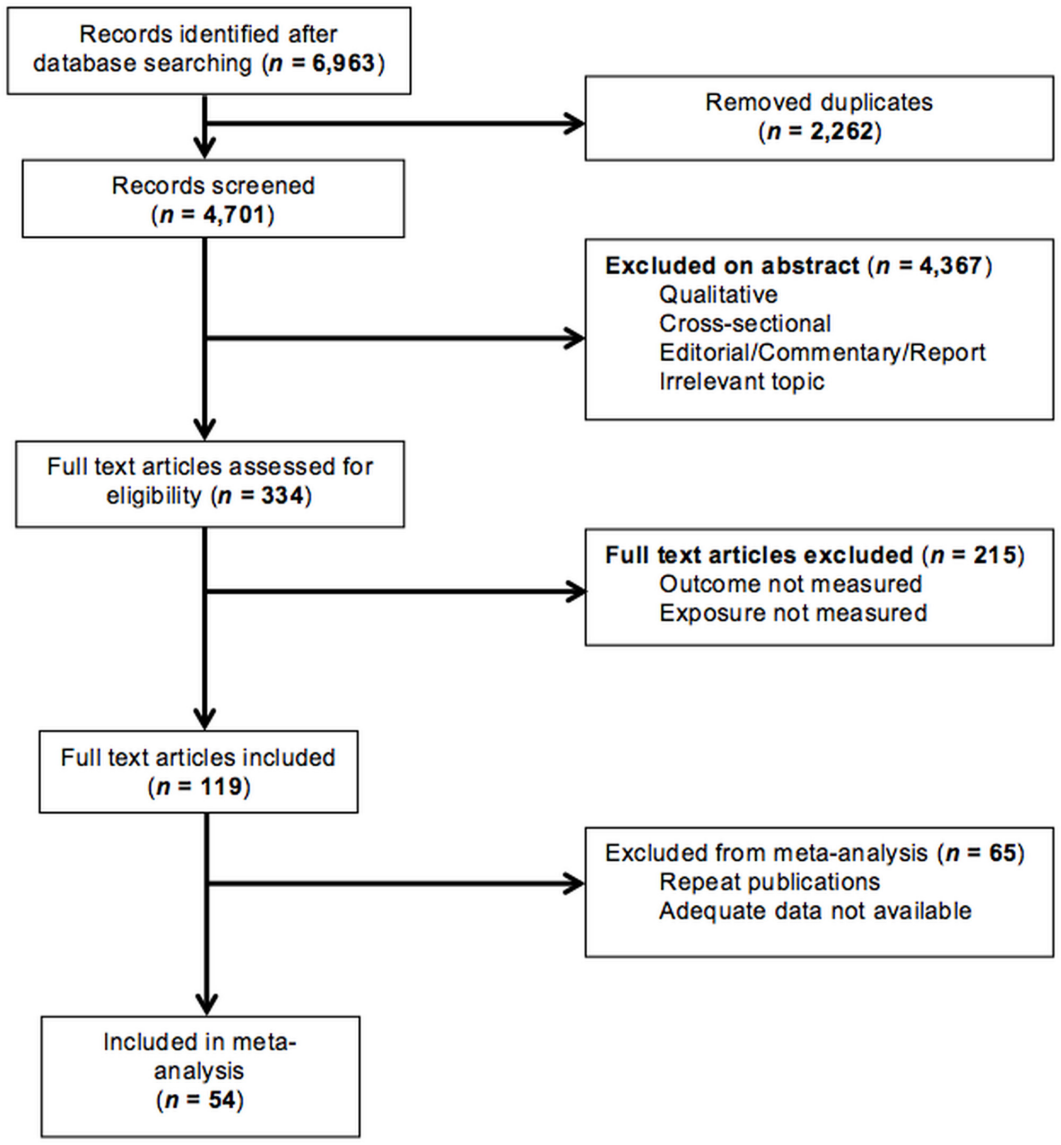
Flow diagram records identified through database searching.

Fig 1 presents a flow diagram of the search strategy. After duplicates were removed the search retrieved 4,701 articles, of which 4,582 were excluded (4,367 on review of abstract and a further 215 after full text assessment). A further 65 articles were omitted. These were 27 repeat publications from the same dataset (see S2 Appendix) and 38 studies where adequate data was not available following contact with authors. Details of these studies are presented in S3 Appendix. Thus data from 54 datasets were included in the analysis.

### Study characteristics

Characteristics of included studies are displayed in S4 Appendix. Eighteen studies were conducted in Europe, twenty-six in North America, eight studies in Asia, and two were conducted in Australia. In the majority of cases, the research design adopted was a cohort study (80% of studies) with study populations of caregivers and dementia care recipient dyads (74% of studies).

There was substantial variation in the types of exposure measures included. Twenty-seven studies measured caregiver burden and of these seventeen studies used the Zarit Burden Interview (ZBI). One study used items from the ZBI and two studies measured burden with the Family Caregiving Burden Inventory (FCBI). A further seven studies used seven different measures of burden (see S4 Appendix).

Ten studies measured carer stress, two of these studies with the Relative Stress Scale. The other eight studies used eight different measures of stress. Six studies measured carer distress: three used the General Health Questionnaire, two used the Neuropsychiatric Inventory Distress scale (NPI-D), and the sixth used a measure from the InterRAI home care assessment tool (see S4 Appendix).

Carer strain was measured in two studies. One study measured carer anxiety using the anxiety scale of the Hospital Anxiety and Depression Scale (HADS). Nineteen studies measured depression, thirteen of these with the Centre for Epidemiologic Studies Depression Scale (CES-D). Three studies used the Geriatric Depression Scale. Three other studies used three different measures of depression.

Eleven studies utilized more than one measure. Six studies measured both carer burden and depression. Two studies measured carer stress and depression. One study measured carer burden and anxiety. One study measured carer stress and distress and one study measure carer distress and depression.

A number of studies referred to the same measure as a measure of burden, stress, or distress. This interchangeability was apparent both within and between studies. For example, in one study the same measure was referred to as both a measure of stress and burden (reference 85, S4 Appendix). The General Health Questionnaire was used to measure ‘stress’ (reference 47; S4 Appendix), ‘distress’ (reference 71; S4 Appendix), and ‘burden’ (reference 102; S4 Appendix).

In forty-two studies the outcome measure was admission to long-term care, in seven studies it was admission to acute care, while in five studies the outcome was admission to both acute and long term care (see S4 Appendix).

### Synthesis of results: Meta-analysis

**Fig 2:**
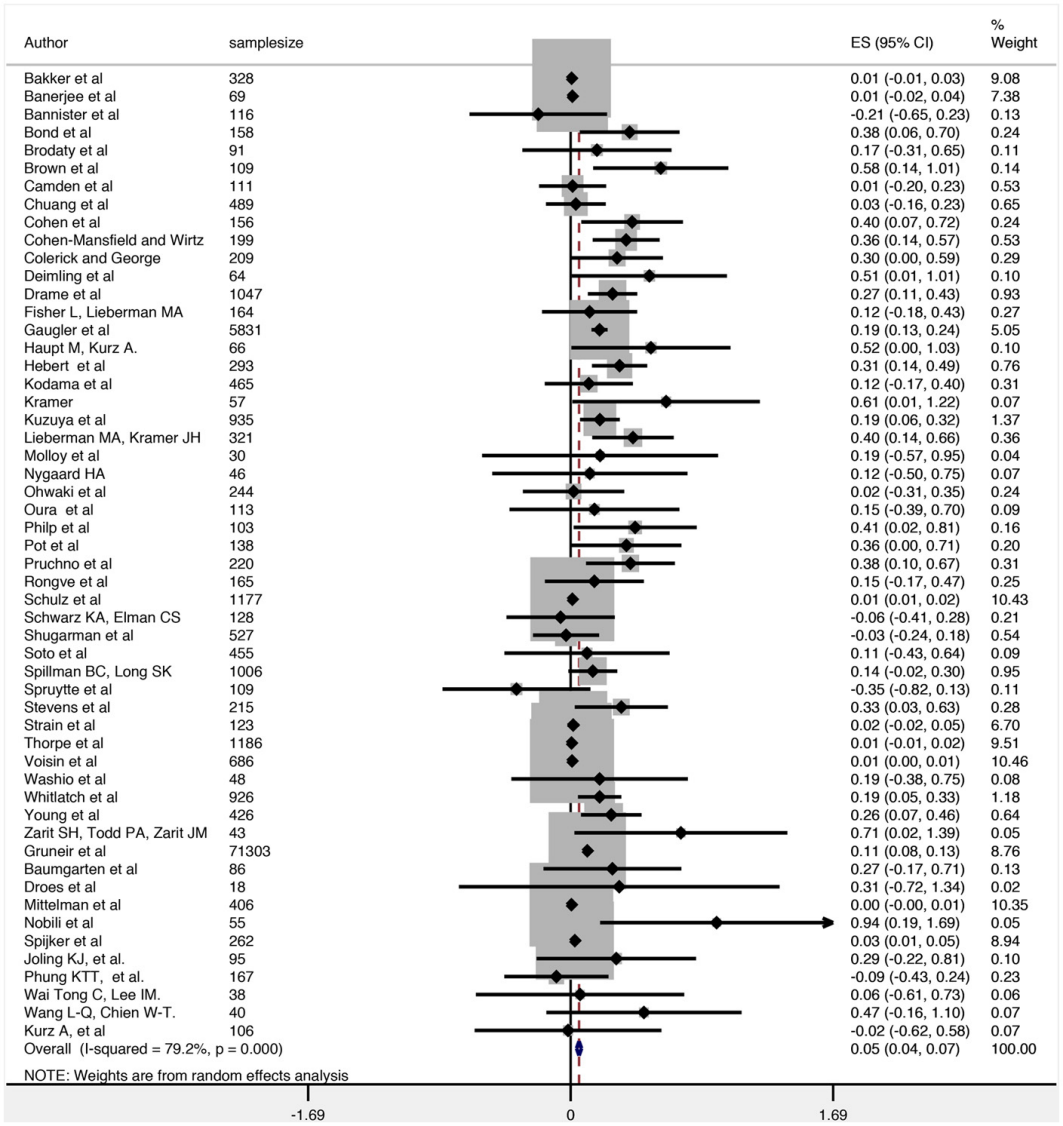
Overall Forest Plot.

As detailed in the methods section, we adopted a hierarchical approach to the exposure measure to estimate the overall effect size. With this approach the meta-analysis found that, while carer stress has a significant effect on subsequent institutionalisation of care recipients, the overall effect size across the 54 studies was negligible according to Cohen’s guidelines (SMD = 0.05, 95% CI = 0.04–0.07) [44]. The forest plot using best estimates from individual studies is displayed in Fig 2.

**Fig 3:**
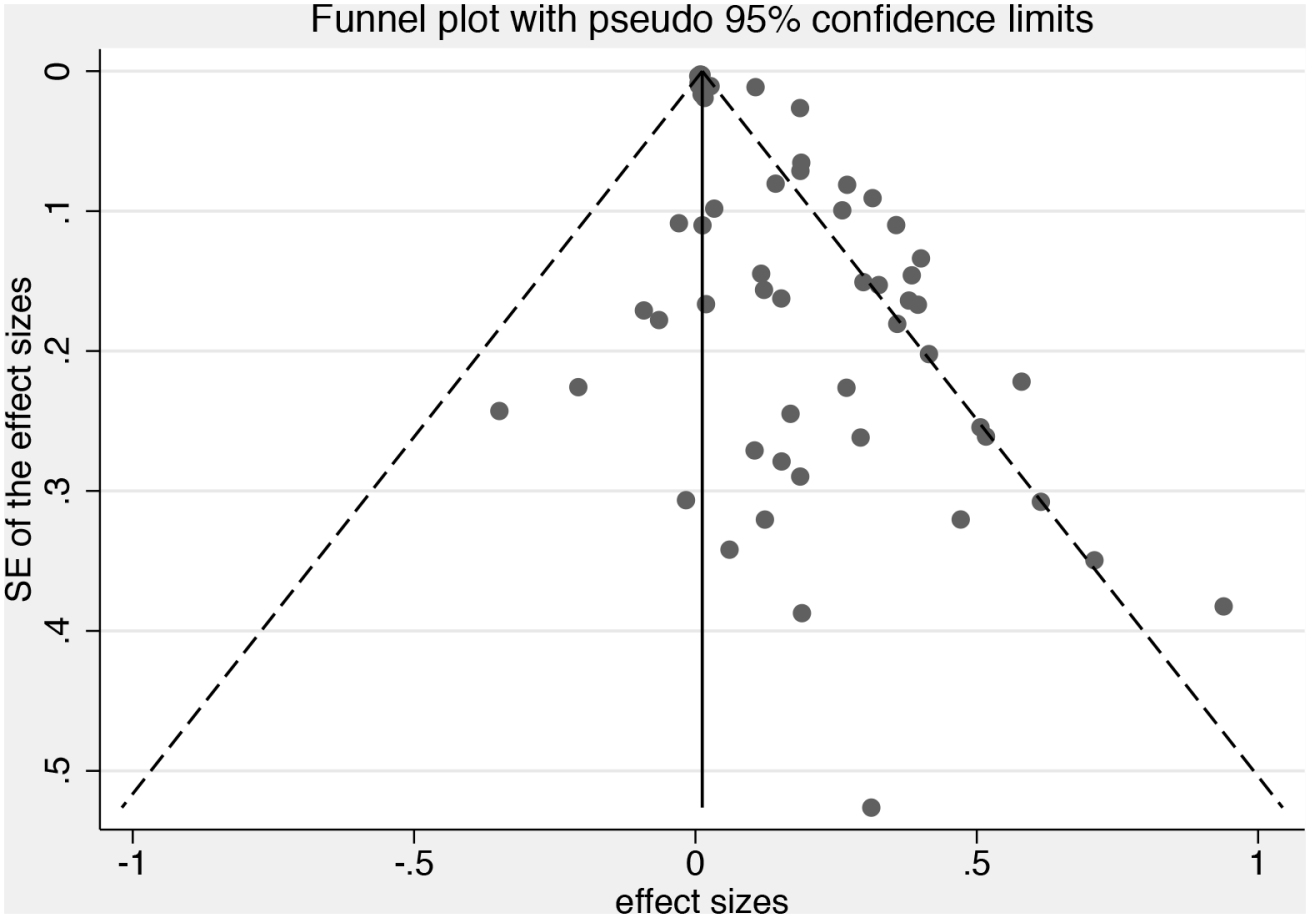
Funnel Plot.

There was evidence of statistically significant heterogeneity (I^2^ = 79.2%; p = <.001) and funnel plot asymmetry (see Fig 3). Furthermore, the Egger’s bias coefficient (bias = 1.45; p = <.001) strongly indicated the presence of asymmetry and publication bias, suggesting small studies overestimate the effect of stress [45].

### Additional analysis: Sensitivity analyses

Summary estimates for each of the sensitivity analyses are displayed in Table 1. To examine the impact of the type of measure on estimates, the sensitivity analysis separated out measures of stress, burden, depression or distress. Where the same scale was used to measure ‘stress’, ‘distress’ and ‘burden’, we applied the original classification by the authors of the scale. For example, the General Health Questionnaire was classified as ‘distress’, an approach also adopted in a similar systematic review [41, 42].

**Table 1:** Sensitivity analyses.

		No. of studies	SMD	95% CI	1^2^	P Value
**Study size**	Small	19	0.28	0.13–0.44	53.5	.003
	Medium	17	0.11	0.05–0.18	57.7	.002
	Large	18	0.05	0.03–0.07	90.1	<.001
**Decade published**	1980s	3	0.40	0.16–0.63	0.0	.494
	1990s	14	0.26	0.18–0.34	0.0	.478
	2000s	23	0.05	0.03–0.07	77.5	<.001
	2010+	14	0.04	0.02–0.07	86.3	<.001
**Quality score**	Group 1: CCAT score < 50%	13	0.26	0.12–0.41	69.5	<.001
	Group 2: CCAT score 50–57%	12	0.21	0.09–0.33	67.4	<.001
	Group 1: CCAT score 58–67%	15	0.05	0.03–0.07	84.1	<.001
	Group 1: CCAT score ≥68%	14	0.05	0.01–0.09	82.6	<.001
**Adjustment**	Unadjusted estimates	44	0.14	0.10–0.18	81.7	<.001
	Adjusted estimates	10	0.01	0.00–0.02	46.6	.051
**Population**	Dementia population	40	0.03	0.02–0.05	72.7	<.001
	Non-Dementia population	14	0.15	0.08–0.22	38.0	.074
**Follow up**	A year or less	26	0.09	0.05–0.14	77.7	<.001
	Over a year	28	0.04	0.02–0.05	77.2	<.001
**Study design**	Cohort	43	0.11	0.08–0.14	82.3	<.001
	Intervention	11	0.01	0.00–0.02	32.9	.136
**Outcome**	Long term care	42	0.06	0.04–0.08	74.2	<.001
	Both acute and long term care	5	0.27	0.13–0.41	0.0	.433
	Acute care	7	0.05	0.00–0.09	92.4	<.001
**Region conducted**	Europe	18	0.02	-0.00–0.05	53.3	.004
	USA	20	0.07	0.04–0.09	83.4	<.001
	Canada	6	0.13	0.04–0.22	81.5	<.001
	Asia	8	0.14	0.05–0.23	0.0	.844
	Australia	2	0.31	0.05–0.58	0.0	.475
**Differences between measures**	Any measure of burden	27	0.07	0.05–0.10	81.1	<.001
	Any measure of stress	10	0.23	0.09–0.38	72.8	<.001
	Any measure of distress	6	0.09	-0.01–0.18	89.5	<.001
	Any measure of depression	19	0.03	0.00–0.05	47.8	.011
	Zarit Burden Interview	17	0.06	0.04–0.09	85.2	<.001
	Any other measure of burden	10	0.19	0.04–0.35	51.9	.028
**Studies with more than one measure**	Burden	6	0.24	0.07–0.42	84.9	<.001
	Depression	6	0.04	-0.00–0.07	71.1	.004

It would appear that the magnitude of the estimate with measures of stress (SMD = 0.23, 95% CI = 0.09–0.38) is higher than that of the estimate with any other exposure measure. This estimate would still be considered ‘small’ according to Cohen’s guidelines [44]. In contrast, the estimate with measures of burden are lower (SMD = 0.07, 95% CI = 0.05–0.10). Estimates with measures of depression fall within this range with confidence intervals which overlap with estimates of burden (SMD = 0.03, 95% CI = 0.00–0.05). However, a number of studies in the sensitivity analysis of the estimate with measures of stress used non-validated measures. We undertook a further sensitivity analysis of those studies measuring stress. Of these studies, for those that used a validated measure of stress (that is the psychometric properties of the scale have been reported), the effect size was approximately half (n = 6; SMD = 0.17, 95% CI = -0.02–0.36) of that of those studies that did not use a validated measure or report if the psychometric properties of the scale had been tested (n = 4; SMD = 0.30, 95% CI = 0.12–0.48). Overall, the number of studies included in these estimates varies, thus it cannot be said with confidence that there is a stronger effect estimate with a particular type of exposure. However, it would appear that irrespective of whether we examine the effect of stress, burden, depression or distress on institutionalisation separately or combined these measures the effect remains significant, but small to negligible according to Cohen’s guidelines [44].

It was also possible to examine estimates solely with measures of burden and stress according to the stress process model [6]. In this case, the size of the effect is also negligible according to Cohen’s guidelines (SMD = 0.06, 95% CI = 0.04–0.08). Similarly, it was possible to examine estimates with measures of psychological distress, as adopted in a similar systematic review [16]. Again, the size of the effect is negligible according to Cohen’s guidelines (SMD = 0.05, 95% CI = 0.01–0.09).

We explored further the impact of the particular exposure measure on effect estimates by comparing studies which applied the Zarit Burden Interview (ZBI) (SMD = 0.06, 95% CI = 0.04–0.09) to those that applied any other measure of burden (SMD = 0.19, 95% CI = 0.04–0.35). While the SMD between the two estimates appears somewhat different, the effect sizes would still be considered small to negligible according to Cohen’s guidelines [44].

Finally, in terms of the effect of different exposure measures, the sensitivity analysis examined differences in estimates within studies that measured both burden (SMD = 0.22, 95% CI = 0.05–0.39) and depression (SMD = 0.03, 95% CI = -0.00–0.07). Again, although the SMD between the two estimates appears quite different, the effect sizes would still be considered small to negligible according to Cohen’s guidelines [44].

**Fig 4:**
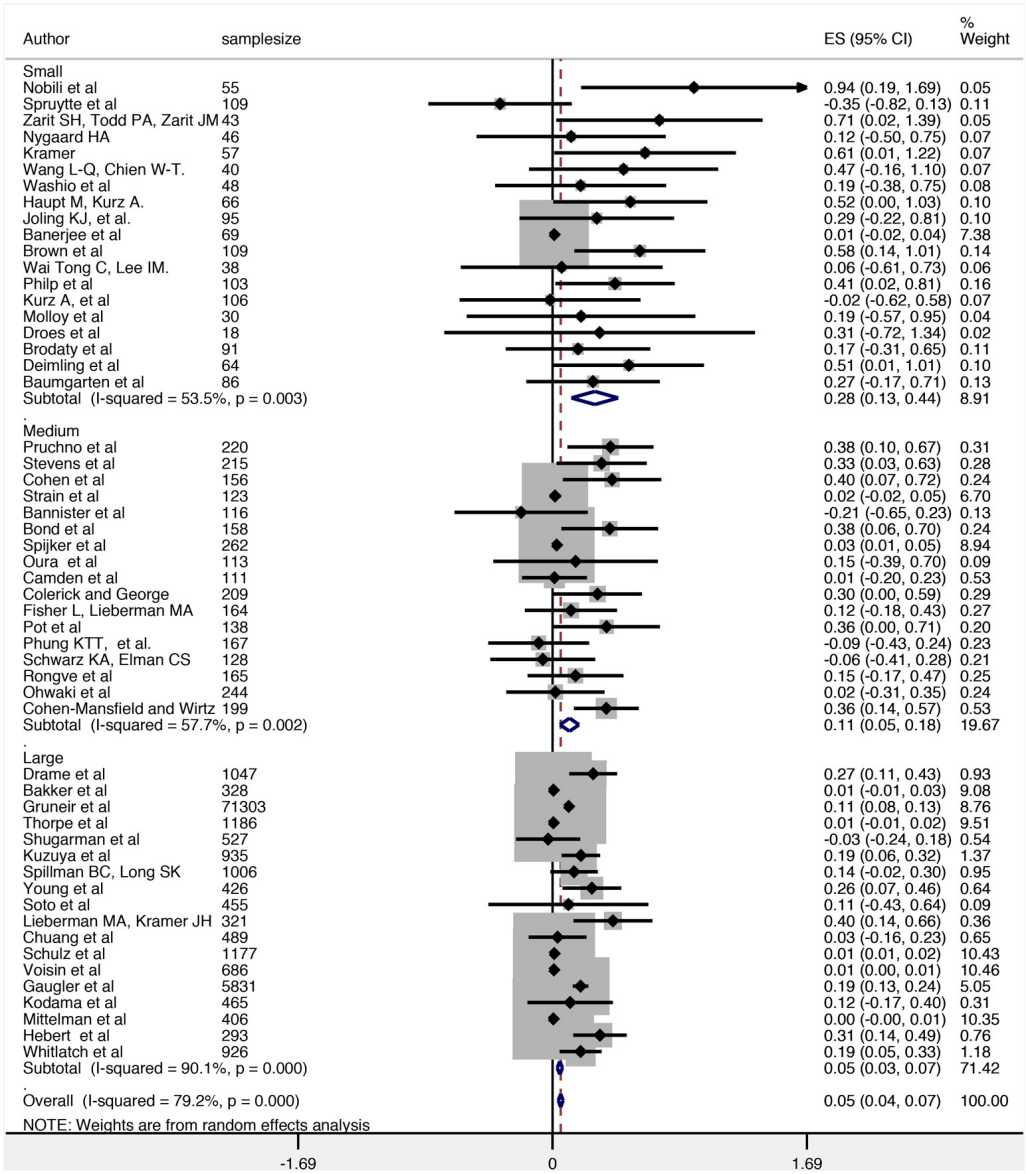
Forest plot by sample size.

To investigate how effect estimates varied according to study size, we grouped studies by tertile. Consistent with the findings of the Egger’s test, there is a notable reduction in the size of the effect for studies with larger sample sizes. For example, the effect estimate in studies with the smallest samples (SMD = 0.28, 95% CI = 0.13–0.44) was higher compared to larger studies (SMD = 0.05, 95% CI = 0.03–0.07). The forest plot with estimates grouped according to their sample size is provided in Fig 4.

The sensitivity analysis also examined estimates according to the decade in which the study was published. It would appear that in each of the last four decades the effect estimates have decreased (1980s: SMD = 0.40, 95% CI = 0.16–0.63; to 2010 and later: SMD = 0.05, 95% CI = 0.02–0.07), as displayed in Table 1.

As presented in S4 Appendix, there was a wide variation in study quality, with CCAT scores ranging from 30% to 95%. To examine the impact of study quality on effect estimates in a meaningful way, we grouped studies by quartiles of CCAT scores. As presented in the table, effect estimates reduce as study quality improves. The effect estimate changes considerably for those studies with unadjusted estimates (SMD = 0.14, 95% CI = .010–0.18) compared to those studies with adjusted estimates (adjusting for factors such as age, sex, type and severity of dementia, spousal carer and ADL rating) (SMD = 0.01, 95% CI = 0.00–0.02). Despite this change, the unadjusted estimate would still be considered a ‘small’ effect size according to Cohen’s guidelines [44].

As displayed in Table 1, the sensitivity analysis also examined the differential effect of the study design on estimates. The estimate with cohort studies appears higher (SMD = 0.11, 95% CI = 0.08–0.14) than that of intervention studies (SMD = 0.01, 95% CI = 0.01–0.02). However, the estimate with cohort studies would still be considered a ‘small’ effect size according to Cohen’s guidelines [44]. In three cases it was not possible to get data on control groups alone, despite contact with authors, therefore a sensitivity analysis examined the impact on estimates when these three studies were excluded, however the effect size remained negligible (SMD = 0.07, 95% CI = 0.05–0.09) [44].

Finally, as displayed in Table 1, the sensitivity analysis included an examination of the impact on estimates depending on the whether the sample was a dementia population or non-dementia population; the period of follow-up; the region in which studies were conducted and the outcome. While estimates vary slightly, in all cases the estimates remain small to negligible according to Cohen’s guidelines.

### Meta-regression

**Fig 5:**
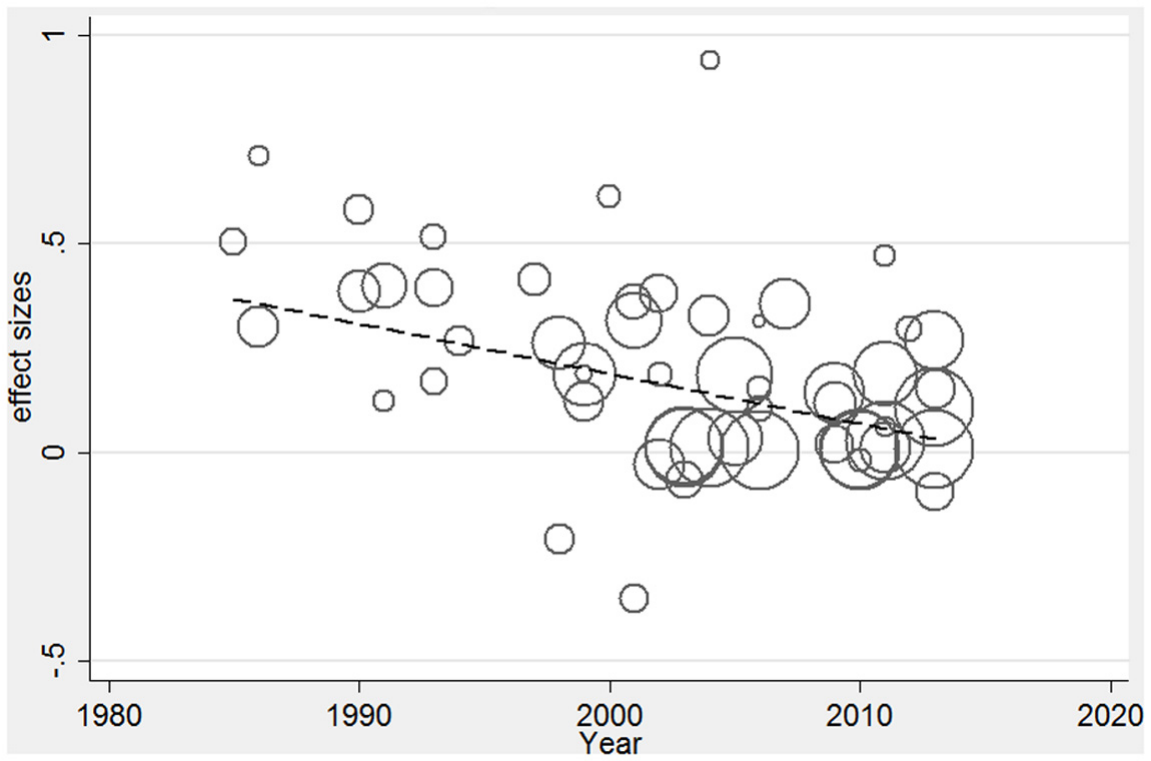
Bubble plot of fitted regression line.

Given significant heterogeneity was found in the overall effect size, we conducted a meta-regression to investigate the contribution of different study characteristics to the level of heterogeneity [46, 47]. Firstly, we examined each study level characteristic individually, as presented in Table 2. We examined the extent to which heterogeneity was related to the year in which studies were published, with year of publication as a continuous variable. From the table, it would appear that there is a significant negative association between the year of publication and the size of the treatment effect. In tandem with this, the year in which studies were published made a substantial contribution to the level of heterogeneity, with the proportion of heterogeneity accounted for at 29.7% (p = <0.001). Fig 5 presents a bubble plot of the fitted regression line with the size of the circles reflecting the relative weight of the study.

**Table 2:** Meta-regression with single covariates.

Study characteristic	Coefficient (SE)	95% CI	Covariate P Value	Joint test for all covariates P Value	Heterogeneity τ^2^	Proportion of between-study variance explained
Year of publication	-0.012 (0.003)	-.0.018–-0.006	<0.001	<0.001	0.009	29.65%
Adjusted	-0.120 (0.047)	-0.215–-0.025	0.014	0.014	0.009	24.54%
Study size	-0.000 (0.000)	-0.000–0.000	0.802	0.802	0.014	-9.50%
Study quality (CCAT %)	-0.134 (0.093)	-0.321–-0.053	0.157	0.157	0.014	-8.97%
Outcome	0.063 (0.053)	-0.043–0.169	0.235	0.235	0.013	-5.30%
Measure—depression	-0.143 (0.084)	-0.311–0.025	0.093	0.463	0.014	-9.87%
Measure—burden	-0.050 (0.067)	-0.185–0.084	0.456			
Measure—distress	-0.118 (0.096)	-0.310–0.075	0.226			
Measure—strain	-0.082 (0.179)	-0.442–0.278	0.650			
Follow-up for 1–2 years	-0.030 (0.070)	-0.172–0.112	0.674	.513	.017	-11.38%
Follow-up or over 2 years	-0.090 (.077)	-0.246–0.066	0.252			

Whether studies included unadjusted or adjusted estimates (adjusting for factors such as age, sex, type and severity of dementia, spousal carer and ADL rating) also made a considerable contribution, accounting for 24.5% of heterogeneity (p = 0.014). As presented in Table 2, we also examined the contribution of study quality, study size, outcome, and type of exposure measure and the period of follow-up, though it would appear these all explained less heterogeneity than would be expected by chance [47].

We entered those variables found to be significant into a meta-regression with multiple covariates, presented in Table 3. In this model, the year in which studies were published and whether studies included adjusted or unadjusted estimates accounted for 46% of heterogeneity. The remaining heterogeneity was small (τ^2^ = 0.007) [47].

**Table 3:** Meta-regression with multiple covariates.

Study characteristic	Coefficient (SE)	95% CI	P
Year of publication	-0.011 (0.003)	-0.017–-0.005	0.001
Adjusted	-0.094 (0.041)	-0.177–-0.011	0.027
**Model Heterogeneity τ** ^**2**^: .007			
**Proportion of between study variance explained:** 45.51%			
**Model P Value:** 0.001			

## Discussion

### Summary of evidence

Overall, the results suggest that while carer stress has a significant effect on subsequent institutionalisation of care recipients, the size of this effect is small to negligible according to Cohen’s guidelines [44]. The sensitivity analysis reinforced this overall effect size. Firstly, in terms of the type of measure, while the estimate for studies that measured stress appeared to be higher than other measures, it was still relatively small. Thus, whether the exposure was a measure of burden, stress, distress, or depression the effect size remained small to negligible. Similarly, we found whether the outcome was acute or long-term care the effect size was negligible. This was also the case when examining estimates solely with measures of stress or burden, according to the stress process model [6], or solely with measures of psychological distress, as also adopted in a similar systematic review [16]. Further, we found the effect size for studies with estimates that had adjusted for other factors to be substantially lower than with studies with un-adjusted estimates. While the un-adjusted estimate is larger than the adjusted, the un-adjusted estimate effect size is still small according to Cohen’s guidelines [44].

Taking the findings together, though carer stress is significant predictor of institutionalisation, the size of this effect suggests that other factors may be more crucial in institutional placement than carer stress. Indeed, when systematically reviewing the association between factors other than carer stress and institutionalisation, a number of studies found functional and cognitive impairment and prior nursing home use to be predictive of admissions to long-term care [23–25, 28]. Prior hospital admissions and duration of previous hospital stay, co-morbidity and polypharmacy have been found to be predictive of admission to acute care [26, 27]. This review suggests that future research should perhaps concentrate on these and other factors in the prediction of institutionalisation as it appears that carer stress is not the most critical determining factor of institutionalisation in older care recipients.

The sensitivity analyses indicated that in each of the last four decades, effect estimates have decreased and are lower in studies with larger samples. This was consistent with both the funnel plot and findings of the Egger’s test, suggesting that small studies over-estimate the size of the effect of carer stress. We also found study quality to have a substantial impact on estimates; with estimates reducing as study quality improves. The findings of the sensitivity analysis were consistent with that of the meta-regression, which found that there was a significant negative association between the year of publication and the size of the treatment effect. Concurrently, results from studies using adjusted estimates also appear to differ from results of studies with un-adjusted estimates.

These findings suggest that, over time, as studies have increased in size, quality has improved, and more factors have been taken into account, the size of the effect has reduced. This suggests that the significant association found between carer stress and institutionalisation in initial studies in this area may have resulted in a belief that higher levels of carer stress can undermine the sustainability of homecare. However, in later years this does not appear to have been critically evaluated, with researchers possibly relying on these initial studies to provide evidence for such contentions [22, 48].

The present findings therefore suggest a need to re-examine this assertion in the literature—while carer stress has a significant effect on subsequent institutionalisation of care recipients, the actual size of this effect is small to negligible. This would suggest that carer stress is not the most critical determining factor of institutionalisation in older care recipients, and that strong statements to the contrary are not based on the evidence available [22]. Such publication bias in psychology is not uncommon. A recent, examination of publication bias in psychology found that barely-significant values were much more frequently reported than values that just failed to reach the conventional threshold for statistical significance [49]. This aversion to publish null results impedes the publication of non-significant replication studies [50, 51], resulting in the continued publication of numerous ‘undead’ theories [51] or what Ioannidis describes as ubiquitous false positive claims [52].

The findings should not be interpreted as undermining the significance of chronic stress on carers, or the importance of RCTs aimed at reducing carer stress. The level of stress experienced by a carer is important both of itself and for its potential impact on the carer, such as impaired psychological well-being and physical health [1], including weakened immunity and wound healing [53, 54]. Indeed, a number of meta-analyses and systematic reviews have concluded that carers are more likely to have poor psychological and physical health outcomes when compared to non-carers [1, 2]. However, as stress appears to have a negligible effect on institutionalisation, it is unlikely that RCTs in this area will have an effect on institutionalisation rates.

### Interchangeability of measures

While carer burden, depression, and stress were the most commonly applied measures, the review found substantial inconsistency across the measurement of psychological morbidity in carers. This inconsistency was apparent in a number of ways. Firstly, the same exposure was assessed with a range of measures. As described earlier, in seven different studies burden was measured seven different ways. Inconsistency was also seen within and between studies. For example, within some studies the same measure was referred to as both a measure of stress and burden, while in different studies the same measure was referred to as a measure of burden, stress, or distress (references 47, 71, 85 and 102, S4 Appendix). The tendency of some researchers to regard measures of carer burden and depression as synonymous with measures of stress has been acknowledged in the literature [4]. This tendency is most apparent in the case of ‘burden’ which has been applied to virtually every dimension of the stress process [10]. Overall this meant that we had to combine these exposures in the present review—however sensitivity analyses did not suggest substantially different effects for these separate measures on care recipient institutionalisation. This confusion in the caregiving literature points to a need for the development of an agreed taxonomy to enable more concise identification of interactions relating to psychological morbidity in the caregiving process.

### Strengths and limitations

Given the lack of clarity and consequential interchangeability in the measurement of psychological morbidity, the search strategy had to incorporate a wide number of exposures that are measured under the umbrella term ‘carer stress’. It could be argued that these exposures represent distinct concepts that when pooled may result in misclassification bias. However, there is theoretical and methodological support to pool the exposure measures, in addition to the practical considerations outlined above given the contradictory literature.

Firstly, Cramer et al., have developed a network approach to mental disorders and comorbidity. According to this approach, symptoms are viewed not as indicators of latent conditions but as components in a network [55]. Borsboom et al., employed this network model to show that half of the symptoms in the DSM-IV are connected [56]. As recommended by Bradburn et al., and Take et al., [36, 37], to enable pooling, the primary measure of effect was the standardized mean difference using a random effects model. By standardization, the results were transformed to a common scale and the random effects model combined data under the assumption that the effect is not fixed between populations but varies around a typical value. Finally, despite the wide variation in exposure measures the meta-regression found that differences in the type of exposure measure explained less heterogeneity than would be expected by chance, providing further support for pooling measures.

The sensitivity analysis examined major aspects of study design that could affect estimates. This included dementia or non-dementia populations, cohort or intervention studies, and the period of follow-up. We found these had a minimal impact on estimates. However, there may still be un-measured confounders. These may include the effect of formal care service utilization or the degree to which access to long-term care varies over time and between health systems—given the availability of beds, differential professional assessments and criteria to access long-term care. We accounted for the region in which studies were published in the sensitivity analysis and found estimates remain small to negligible. However, it would not be appropriate to give too much weight to this comparison given health system variation within and between countries. This would be more appropriate in future research.

Finally, the lack of access to unpublished data could also be regarded as a limitation. However, both the funnel plot and the Egger’s bias coefficient strongly indicated the presence of publication bias, suggesting small studies overestimate the effect of stress [45]. Therefore, had it been possible to access unpublished data, it is likely that the effect sizes found would have been even smaller than what was found.

### Future research

The findings suggest the need to critically review the definition of carer stress, to offer some clarity around the terminology used and consolidate measures to enable more precise identification of the interactions relating to psychological morbidity in the caregiving process.

Future research should perhaps concentrate on other factors found to be associated with institutionalisation, such as the characteristics of the care recipient. This research could be expanded to account for the impact of the health system on long-term care provision. In tandem with this, as a carer is ultimately responsible for the decision to yield care, yet carer stress appears to have a small to negligible effect on institutional placement, qualitative work could be employed to enable more in-depth examination of the impact of carer stress on the decision to yield care.

## Conclusion

The results of this review suggest that while carer stress has a significant effect on subsequent institutionalisation of care recipients, the size of this effect is small to negligible. The sensitivity analysis reinforced the effect size; irrespective of the type of measures used, carer stress has small to negligible effect on subsequent institutionalisation. The results also highlight the problematic nature of the contradictory literature. That is in terms of the interchangeability in the measurement of stress.

Results of this systematic review are at odds with the strong contention that higher levels of carer stress could undermine the sustainability of home care [20–22], and suggest that publication bias, or at least small study effects, have contributed to this belief. The findings should not be interpreted as undermining the significance of chronic stress on carers. As referred to above, a number of meta-analyses and systematic reviews have concluded that carers are more likely to have poor psychological and physical health outcomes when compared to non-carers. However, the size of the effect of carer stress on subsequent institutionalisation suggests that other factors may be more crucial in institutional placement than carer stress.

## Supporting Information

S1 Appendix Medline (OVID) search strategy. (DOCX)

S2 Appendix Repeat publications from the same dataset. (DOCX)

S3 Appendix Characteristics of studies for whom adequate data was not available. (DOCX)

S4 Appendix Characteristics of included studies. (DOCX)

S5 Appendix PRISMA Checklist. (DOCX)

S1 Dataset (XLS)
