# Supplementary material for: Systematic Review and Meta-Analysis of the Impact of Carer Stress on Subsequent Institutionalisation of Community-Dwelling Older People
Source: PLoS One. 2015 Jun 2;10(6):e0128213. doi: 10.1371/journal.pone.0128213 (PMC4452721; doi:10.1371/journal.pone.0128213)
Supplement: S1 Appendix — (DOCX) [file pone.0128213.s001.docx]

## S1 Appendix: Medline (OVID) search strategy

#1 (carer$ or caregiver$).ab,kf,sh,ti.

#2 (dementia or alzheimer$ or elderly or aged).ab,kf,sh,ti.

#3 (stress or burden or burnout or distress or anxiety or depression or strain).ab,kf,sh,ti.

#4 (nursing home$ or Institutionali#ation or long term care or long term utilisation or care home or homes for the aged or acute care or hospitali#ation or (hospital admission or hospital readmission) or emergency department or emergency services department or (accident and emergency)).ab,ti,kf,sh.

#5 #1 AND #2 AND #3 AND #4
