# Supplementary material for: Systematic Review and Meta-Analysis of the Impact of Carer Stress on Subsequent Institutionalisation of Community-Dwelling Older People
Source: PLoS One. 2015 Jun 2;10(6):e0128213. doi: 10.1371/journal.pone.0128213 (PMC4452721; doi:10.1371/journal.pone.0128213)
Supplement: S2 Appendix — (DOCX) [file pone.0128213.s002.docx]

**S2 Appendix: Repeat publications from the same dataset**

| **Table 1 Repeat publications from the same dataset** | | |
| --- | --- | --- |
| **Author** | **Year of publication** | **Title** |
| Mausbach et al.[[1](#_ENREF_1)] | 2004 | Ethnicity and time to institutionalization of dementia patients: a comparison of Latina and Caucasian female family caregivers |
| de Vugt et al.[[2](#_ENREF_2)] | 2005 | A prospective study of the effects of behavioral symptoms on the institutionalization of patients with dementia |
| Benoit et al.[[3](#_ENREF_3)] | 2005 | One-year longitudinal evaluation of neuropsychiatric symptoms in Alzheimer's disease. The REAL.FR study |
| Cohen-Mansfield and Wirtz.[[4](#_ENREF_4)] | 2009 | The reasons for nursing home entry in an adult day care population: Caregiver reports versus regression results |
| Cohen-Mansfield and Wirtz.[[5](#_ENREF_5)] | 2011 | Predictors of entry to the nursing home: does length of follow-up matter? |
| Gaugler et al.[[6](#_ENREF_6)] | 2000 | Predictors of institutionalization of cognitively impaired elders: Family help and the timing of placement |
| Gaugler et al.[[7](#_ENREF_7)] | 2003 | Caregiving and institutionalization of cognitively impaired older people: utilizing dynamic predictors of change |
| Gaugler et al.[[8](#_ENREF_8)] | 2005 | The Effects of Duration of Caregiving on Institutionalization |
| Nikzad-Terhune et al.[[9](#_ENREF_9)] | 2010 | Do trajectories of at-home dementia caregiving account for burden after nursing home placement? A growth curve analysis |
| Gaugler et al.[[10](#_ENREF_10)] | 2005 | The longitudinal effects of early behavior problems in the dementia caregiving career |
| Gaugler et al.[[11](#_ENREF_11)] | 2006 | Predictors of institutionalization in Latinos with dementia |
| Gaugler et al.[[12](#_ENREF_12)] | 2004 | Predictors of Nursing Home Placement in African Americans with Dementia |
| Balardy et al.[[13](#_ENREF_13)] | 2005 | Predictive factors of emergency hospitalisation in Alzheimer's patients: Results of one-year follow-up in the REAL.FR cohort |
| Rolland et al.[[14](#_ENREF_14)] | 2007 | Wandering behavior and Alzheimer disease. The REAL.FR prospective study |
| Winslow and Carter.[[15](#_ENREF_15)] | 1999 | Patterns of burden in wives who care for husbands with dementia |
| Yaffe et al.[[16](#_ENREF_16)] | 2002 | Patient and caregiver characteristics and nursing home placement in patients with dementia |
| Belle et al.[[17](#_ENREF_17)] | 2006 | Enhancing the quality of life of dementia caregivers from different ethnic or racial groups: A randomized, controlled trial |
| Dröes et al[[18](#_ENREF_18)] | 2004 | Effect of Meeting Centres Support Program on feelings of competence of family carers and delay of institutionalization of people with dementia |
| Gaugler et al.[[19](#_ENREF_19)] | 2005 | Early community-based service utilization and its effects on institutionalization in dementia caregiving |
| Kuzuya et al.[[20](#_ENREF_20)] | 2012 | Day-care service use is a risk factor for long-term care placement in community-dwelling dependent elderly |
| Mittelman et al.[[21](#_ENREF_21)] | 1993 | An intervention that delays institutionalization of Alzheimer's disease patients: Treatment of spouse-caregivers |
| Hirdes et al.[[22](#_ENREF_22)] | 2008 | The Method for Assigning Priority Levels (MAPLe): A new decision-support system for allocating home care resources |
| Gaugler et al.[[23](#_ENREF_23)] | 2010 | The effects of incident and persistent behavioral problems on change in caregiver burden and nursing home admission of persons with dementia |
| Morales-Asencio et al.[[24](#_ENREF_24)] | 2008 | Effectiveness of a nurse-led case management home care model in Primary Health Care. A quasi-experimental, controlled, multi-centre study |
| Montgomery and Kosloski.[[25](#_ENREF_25)] | 1994 | A longitudinal analysis of nursing home placement for dependent elders cared for by spouses vs adult children |
| Brodaty and Gresham.[[26](#_ENREF_26)] | 1989 | Effect of a training programme to reduce stress in carers of patients with dementia |

# References

1. Mausbach BT, Coon DW, Depp C, Rabinowitz YG, Wilson-Arias E, Kraemer HC, et al. Ethnicity and time to institutionalization of dementia patients: a comparison of Latina and Caucasian female family caregivers. Journal of the American Geriatrics Society. 2004;52(7):1077-84.

2. de Vugt ME, Stevens F, Aalten P, Lousberg R, Jaspers N, Verhey FR. A prospective study of the effects of behavioral symptoms on the institutionalization of patients with dementia. International Psychogeriatrics. 2005;17(4):577-89.

3. Benoit M, Robert PH, Staccini P, Brocker P, Guerin O, Lechowshi L, et al. One-year longitudinal evaluation of neuropsychiatric symptoms in Alzheimer's disease. The REAL.FR study. Journal of Nutrition, Health and Aging. 2005;9(2):95-9.

4. Cohen-Mansfield J, Wirtz PW. The reasons for nursing home entry in an adult day care population: Caregiver reports versus regression results. Journal of Geriatric Psychiatry and Neurology. 2009;22(4):274-81.

5. Cohen-Mansfield J, Wirtz PW. Predictors of entry to the nursing home: does length of follow-up matter? Archives of gerontology and geriatrics. 2011;53(3):309-15.

6. Gaugler JE, Edwards AB, Femia EE, Zarit SH, Stephens MA, Townsend A, et al. Predictors of institutionalization of cognitively impaired elders: family help and the timing of placement. The journals of gerontology Series B, Psychological sciences and social sciences. 2000;55(4):P247-55.

7. Gaugler JE, Kane RL, Kane RA, Clay T, Newcomer R. Caregiving and institutionalization of cognitively impaired older people: utilizing dynamic predictors of change. The Gerontologist. 2003;43(2):219-29.

8. Gaugler JE, Kane RL, Kane RA, Clay T, Newcomer RC. The Effects of Duration of Caregiving on Institutionalization. The Gerontologist. 2005;45(1):78-89.

9. Nikzad-Terhune KA, Anderson KA, Newcomer R, Gaugler JE. Do trajectories of at-home dementia caregiving account for burden after nursing home placement? A growth curve analysis. Social Work in Health Care. 2010;49(8):734-52.

10. Gaugler JE, Kane RL, Kane RA, Newcomer R. The longitudinal effects of early behavior problems in the dementia caregiving career. Psychology and Aging. 2005;20(1):100-16.

11. Gaugler JE, Kane RL, Kane RA, Newcomer R. Predictors of institutionalization in Latinos with dementia. Journal Of Cross-Cultural Gerontology. 2006;21(3-4):139-55.

12. Gaugler JE, Leach CR, Clay T, Newcomer RC. Predictors of Nursing Home Placement in African Americans with Dementia. Journal of the American Geriatrics Society. 2004;52(3):445-52.

13. Balardy L, Voisin T, Cantet C, Vellas B. Predictive factors of emergency hospitalisation in Alzheimer's patients: Results of one-year follow-up in the REAL.FR cohort. Journal of Nutrition, Health and Aging. 2005;9(2):112-6.

14. Rolland Y, Andrieu S, Cantet C, Morley JE, Thomas D, Nourhashemi F, et al. Wandering behavior and Alzheimer disease. The REAL.FR prospective study. Alzheimer Disease and Associated Disorders. 2007;21(1):31-8.

15. Winslow BW, Carter P. Patterns of burden in wives who care for husbands with dementia. Nursing Clinics of North America. 1999;34(2):275-87.

16. Yaffe K, Fox P, Newcomer R, Sands L, Lindquist K, Dane K, et al. Patient and caregiver characteristics and nursing home placement in patients with dementia. JAMA : the journal of the American Medical Association. 2002;287(16):2090-7.

17. Belle SH, Burgio L, Burns R, Coon D, Czaja SJ, Gallagher-Thompson D, et al. Enhancing the quality of life of dementia caregivers from different ethnic or racial groups: a randomized, controlled trial. Annals of internal medicine [Internet]. 2006; 145(10):[727-38 pp.]. Available from: <http://onlinelibrary.wiley.com/o/cochrane/clcentral/articles/841/CN-00573841/frame.html>.

18. Dröes R, Breebaart E, Meiland FJ, Van Tilburg W, Mellenbergh GJ. Effect of Meeting Centres Support Program on feelings of competence of family carers and delay of institutionalization of people with dementia. Aging & Mental Health. 2004;8(3):201-11.

19. Gaugler JE, Kane RL, Kane RA, Newcomer R. Early community-based service utilization and its effects on institutionalization in dementia caregiving. Gerontologist. 2005;45(2):177-85.

20. Kuzuya M, Izawa S, Enoki H, Hasegawa J. Day-care service use is a risk factor for long-term care placement in community-dwelling dependent elderly. Geriatrics & gerontology international. 2012;12(2):322-9.

21. Mittelman MS, Ferris SH, Steinberg G, Shulman E, Mackell JA, Ambinder A, et al. An intervention that delays institutionalization of Alzheimer's disease patients: Treatment of spouse-caregivers. The Gerontologist. 1993;33(6):730-40.

22. Hirdes JP, Poss JW, Curtin-Telegdi N. The Method for Assigning Priority Levels (MAPLe): A new decision-support system for allocating home care resources. Bmc Medicine. 2008;6:11.

23. Gaugler JE, Wall MM, Kane RL, Menk JS, Sarsour K, Johnston JA, et al. The effects of incident and persistent behavioral problems on change in caregiver burden and nursing home admission of persons with dementia. Medical care. 2010;48(10):875-83.

24. Morales-Asencio JM, Gonzalo-Jimenez E, Martin-Santos FJ, Morilla-Herrera JC, Celdraan-Maas M, Carrasco AM, et al. Effectiveness of a nurse-led case management home care model in Primary Health Care. A quasi-experimental, controlled, multi-centre study. BMC Health Services Research. 2008;8.

25. Montgomery RJ, Kosloski K. A longitudinal analysis of nursing home placement for dependent elders cared for by spouses vs adult children. J Gerontol. 1994;49(2):S62-S74.

26. Brodaty H, Gresham M. Effect of a training programme to reduce stress in carers of patients with dementia. BMJ (Clinical research ed) [Internet]. 1989; 299(6712):[1375-9 pp.]. Available from: <http://onlinelibrary.wiley.com/o/cochrane/clcentral/articles/672/CN-00064672/frame.html>.
