# Supplementary material for: Systematic Review and Meta-Analysis of the Impact of Carer Stress on Subsequent Institutionalisation of Community-Dwelling Older People
Source: PLoS One. 2015 Jun 2;10(6):e0128213. doi: 10.1371/journal.pone.0128213 (PMC4452721; doi:10.1371/journal.pone.0128213)
Supplement: S3 Appendix — (DOCX) [file pone.0128213.s003.docx]

**S3 Appendix: Characteristics of studies for whom adequate data was not available**

| **Table 2 Characteristics of studies for whom adequate data was not available** | | | | | | |
| --- | --- | --- | --- | --- | --- | --- |
| **Author; country** | **Year of publication** | **Research design** | **Study population; % male care recipients** | **Sample size: baseline; follow-up** | **Exposure measure** | **Outcome measure** |
| Arai et al.[[1](#_ENREF_1)]; Japan | 2001 | 1 year cohort | Certifiably registered disabled elderly who lived at home with their caregivers. % men not provided | 70; 65 | CES-D and Zarit Burden Interview | Discontinued caring. |
| D'Souza et al.[[2](#_ENREF_2)]; United States | 2009 | 4 year cohort | Individuals enrolled in the Michigan Home and Community-Based Services (HCBS) waiver program for elderly and disabled adults and their carers. 27% | 108,914; 108,914 | Caregiver burden was defined as indication on either of two variables from the target MDS-HC: “primary caregiver expresses feelings of distress, anger, or depression” or “ a caregiver is unable to continue in caretaking activities. ” | Emergency room (ER) use, hospitalisation and permanent nursing home placement |
| Froelich, et al.[[3](#_ENREF_3)]; 12 European countries | 2009 | 2 year cohort | Dementia dyads. 37.5% | 2,288; 1,382 | Zarit burden interview. Distress as part of the NPI-D | Admission to permanent institutionalisation |
| Hooley et al.[[4](#_ENREF_4)]; Canada | 2005 | 6 month cohort | Outpatients attending the Heart Function Clinic at a large tertiary care hospital and their carers. 72% | 50; 50 | Zarit burden interview | Hospitalisation |
| Okura et al.[[5](#_ENREF_5)]; United States | 2011 | 5 year cohort | Mix dementia and non-dementia dyads. % men not provided | 453; 453 | Distress with the NPI-D | Nursing Home Placement |
| Schur D, Whitlatch CJ.[[6](#_ENREF_6)]; United States; | 2003 | 6 month non-randomised intervention | Elders with mental and or physical impairments and their carers. 45% | 127; 127 | Carer stress measured with the Revised version of the Memory and Behaviour Problems Checklist. Depression measured with the abridged version of the CES-D. | Institutionalisation |
| Andren S, Elmstahl S.[[7](#_ENREF_7)]; Sweden | 2008 | 5 year non randomised intervention study | Dementia dyads. 23% | 192; 192 | Burden with the caregiver burden scale [[8](#_ENREF_8)] . | Nursing Home Placement |
| Cho etal.[[9](#_ENREF_9)]; United States | 2009 | 2 year non randomised intervention study | Dementia dyads. 0% | 371; 371 | Depression with CES-D | Nursing Home Placement |
| McMillan et al.[[10](#_ENREF_10)]; United States | 2013 | 5 week intervention | Community dwelling adults with heart disease and an identified family caregiver. 65% | 30; 21 | Carer stress with MSAS-HF. Depression measure with the depression subscale from the Profile of Mood States (POMS). | Hospitalisation and Emergency Room visits |
| Menn et al.[[11](#_ENREF_11)]; Germany | 2012 | 4 year RCT | Dementia dyads. 33% | 171; 134 | Burden Scale for Family Caregivers (BSFC). | Nursing Home Placement |
| Morales-Asencio et al.[[12](#_ENREF_12)]; Spain | 2009 | 34 month cohort | Community dwelling Patients and caregivers who initiated a Home Care Programme. 42% | 118; 118 | Zarit burden interview | Institutionalisation |
| Thomas R.[[13](#_ENREF_13)]; Canada | 2007 | 4 year RCT | Community dwelling people 75 or over living in their own homes not receiving formal home care, able to identify an informal caregiver. 32% | 520; 307 | Zarit burden interview | Institutionalisation |
| Callahan et al.[[14](#_ENREF_14)]; United States | 2006 | 18 month RCT | Alzheimer's disease dyads. Usual care % men: 73%. Intervention % men: 61% | 153; 153 | Stress with the NPI-D | Hospitalisation and nursing home placement |
| Challis D et al.[[15](#_ENREF_15)]; UK | 2004 | 1 year RCT | Non-dementia dyads. Usual care: 28%. Intervention: 26% | 256; 256 | Stress and burden with the Social Behaviour Assessment Schedule modified for use with carers of older people. | Nursing home placement |
| Crotty, M. et al.[[16](#_ENREF_16)]; Australia | 2008 | 6 month RCT | Hospitalised patients referred for ambulatory rehabilitation. 48% | 98; 98 | Carer stress with Carer Strain Index | Hospital readmission and transfer to residential care |
| Forster A, et al.[[17](#_ENREF_17)]; UK | 2009 | 12 month RCT | Patients with a disabling stroke and their carers. Usual care: 48%. Intervention: 43% | 265; 242 | Carer stress with the general health questionnaire and strain with carer strain index | Hospital readmission |
| Montgomery RJ, Borgatta EF.[[18](#_ENREF_18)]; United States | 1989 | 20 month RCT | Impaired elderly persons and caregivers. 33% | 541; 541 | Burden with nine-item scale developed by Montgomery et al. [[19](#_ENREF_19)] | Nursing home placement |
| Oktay JS, Volland PJ.[[20](#_ENREF_20)]; United States | 1990 | 12 month quasi-experimental intervention | Caregivers and frail elderly discharged from hospital. 39% | 191; 112 | Stress with General Health Questionnaire | Hospital and nursing home days |
| Ryynanen OP, et al.[[21](#_ENREF_21)]; Finland | 2013 | 2 year RCT | Mix dementia and non-dementia dyads. Usual care: 70%. Intervention: 63%. | 135; 113 | Zung's depression scale | Institutionalisation |
| Stewart M, et al.[[22](#_ENREF_22)]; Canada | 2010 | 2 year non-randomised intervention | Mix dementia and non-dementia dyads. Usual care: 48.8%. Intervention: 48.8% | 164; 164 | Zarit burden interview | Emergency department (ED) visits |
| Tibaldi V, et al.[[23](#_ENREF_23)]; Italy | 2007 | 2 year RCT | Dementia dyads. 33% | 82; 25 | Stress measured with the Relative Stress Scale | Institutionalisation |
| Tourigny A, et al.[[24](#_ENREF_24)]; Canada | 2004 | 3 year quasi-experimental intervention | Frail elderly living in a semi-urban community. Intervention: 30%. Control: 34% | 482; 218 | Zarit burden interview | ED visits and/or hospitalisations |
| Vellas B, et al.[[25](#_ENREF_25)]; 11 European countries | 2012 | 2 year cohort | Alzheimer's disease dyads. 35% | 1306; 797 | Zarit burden interview | Hospital admission and admission to nursing home |
| Vincent C, et al.[[26](#_ENREF_26)]; Canada | 2006 | 9 month cohort | Frail elderly living at home and their carers. 8% | 38; 38 | Caregiver burden scale [[8](#_ENREF_8)] | Hospitalisation |
| Whitlatch CJ, et al.[[27](#_ENREF_27)]; United States | 1995 | 1 year quasi-experimental intervention | Dementia dyads. % men not provided | 132; 90 | Burden measured with subscales of the Zarit burden interview | Institutionalisation |
| Lim WK, et al.[[28](#_ENREF_28)]; Australia | 2003 | 6 month RCT | Patients aged 65 years and over who required community services after discharge and their carer. Intervention: 40%. Control: 43%. | 654; 598 | Caregiver strain index | Hospital readmission |
| Lindpaintner LS, et al.[[29](#_ENREF_29)]; Switzerland | 2013 | 1 month RCT | Community-dwelling patients at high risk for adverse medicine events who showed additional evidence of vulnerability and their carers. 43% | 60; 60 | Caregiver strain index | Hospital readmission |
| Kalra L, et al.[[30](#_ENREF_30)]; UK | 2004 | 1 year RCT | Stroke patients and their care givers. 53% | 300; 268 | Caregiver strain index | Institutionalisation |
| Mohide E, et al.[[31](#_ENREF_31)]; Canada | 1990 | 6 month RCT | Dementia dyads. Intervention: 30%. Control: 27% | 60; 42 | Depression with CES-D | Institutionalisation |
| Weinberger M, et al.[[32](#_ENREF_32)]; United States | 1993 | 6 month RCT | Dementia dyads. Intervention: 35%. Control: 41% | 264; 227 | Assessed carer stress but does not detail how this was measured | Hospital admission and admission to nursing home |
| Wilkinson et al.[[33](#_ENREF_33)]; UK | 1997 | 5 year cohort | Stroke patients and their care givers. 53% | 291; 106 | Caregiver strain index | Institutionalisation |
| Hughes SL, et al.[[34](#_ENREF_34)]; United States | 2000 | 1 year RCT | Disabled elderly and their carers. Intervention: 97%. Control: 96% | 1966; 667 | Burden with scale developed by Montgomery et al. [[19](#_ENREF_19)] | Hospital readmission |
| Jette et al.[[35](#_ENREF_35)]; United States | 1995 | 6 year cohort | Disabled elderly and their carers. 23% | 634; 586 | Burden with measures developed for the study. Three measures used: 1. Impact on personal time and physical health; 2. Impact on family relationships; and 3. Impact on decision to work and/or work schedule. | Nursing Home Entry |
| Tsuji et al.[[36](#_ENREF_36)]; United States | 1995 | 5 year chart review | Frail older patients receiving formal home care services and their carers. % men not provided | 334; 334 | Stress due to caregiving (not clear if this was a composite measure) | Nursing Home Placement |
| Gilley et al.[[37](#_ENREF_37)]; United States | 2005 | 3 year cohort | Alzheimer's Disease dyads. 32% | 396; 396 | Perceived burden measured with the 10-item subjective caregiving burden scale [[38](#_ENREF_38)]. | Institutionalisation |
| Aneshensel et al.[[39](#_ENREF_39)]; United States | 1993 | 2 year cohort | Dementia dyads. % men not provided | 555; 494 | Measured stress based on Pearlin's model. Primary stressors: cognitive status of care recipient; problematic behaviour; ADLs and IADLs; role overload; relational deprivation; and patient resistence to help. Secondary stressors: family conflict; conflicting demands of job and caregiving; economic strain; role captivity. [[40](#_ENREF_40)] | Institutionalisation. |
| McCann et al.[[41](#_ENREF_41)]; United States | 2005 | 4 year intervention | Alzheimer's Disease dyads. 32% | 298; 298 | Perceived burden measured with the 10-item subjective caregiving burden scale [[38](#_ENREF_38)]. | Nursing Home Placement |
| Kurasawa et al.[[42](#_ENREF_42)]; Japan | 2012 | 2 year cohort | Disabled elderly and their carers. 35% | 133; 90 | Burden with the Zarit burden interview. Depression with CES-D. | Institutionalisation |

# References

1. Arai Y, Sugiura M, Washio M, Miura H, Kudo K. Caregiver depression predicts early discontinuation of care for disabled elderly at home. Psychiatry Clin Neurosci. 2001;55(4):379-82.

2. D'Souza JC, James ML, Szafara KL, Fries BE. Hard times: the effects of financial strain on home care services use and participant outcomes in Michigan. Gerontologist. 2009;49(2):154-65.

3. Froelich L, Andreasen N, Tsolaki M, Foucher A, Kavanagh S, Baelen BV, et al. Long-term treatment of patients with Alzheimer's disease in primary and secondary care: results from an international survey. Curr Med Res Opin. 2009;25(12):3059-68.

4. Hooley PJ, Butler G, Howlett JG. The relationship of quality of life, depression, and caregiver burden in outpatients with congestive heart failure. Congest Heart Fail. 2005;11(6):303-10.

5. Okura T, Plassman BL, Steffens DC, Llewellyn DJ, Potter GG, Langa KM. Neuropsychiatric symptoms and the risk of institutionalization and death: the aging, demographics, and memory study. Journal of the American Geriatrics Society. 2011;59(3):473-81.

6. Schur D, Whitlatch CJ. Circumstances leading to placement: a difficult caregiving decision. Lippincotts Case Manag. 2003;8(5):187-97.

7. Andren S, Elmstahl S. Effective psychosocial intervention for family caregivers lengthens time elapsed before nursing home placement of individuals with dementia: A five year follow-up study. International Psychogeriatrics. 2008;20(6):1177-92.

8. Elmstahl S, Malmberg B, Annerstedt L. Caregiver's burden of patients 3 years after stroke assessed by a novel caregiver burden scale. Archives of Physical Medicine and Rehabilitation. 1996;77(2):177-82.

9. Cho S, Zarit SH, Chiriboga DA. Wives and daughters: the differential role of day care use in the nursing home placement of cognitively impaired family members. Gerontologist. 2009;49(1):57-67.

10. McMillan SC, Small BJ, Haley WE, Zambroskio C, Buck HG. The COPE Intervention for Caregivers of Patients With Heart Failure. J Hosp Palliat Nurs. 2013;15(4):196-206.

11. Menn P, Holle R, Kunz S, Donath C, Lauterberg J, Leidl R, et al. Dementia care in the general practice setting: a cluster randomized trial on the effectiveness and cost impact of three management strategies. Value in Health. 2012;15(6):851-9.

12. Morales-Asencio JM, Morilla-Herrera JC, Martin-Santos FJ, Gonzalo-Jimenez E, Cuevas-Fernandez-Gallego M, Bonill de Las Nieves C, et al. The association between nursing diagnoses, resource utilisation and patient and caregiver outcomes in a nurse-led home care service: longitudinal study. International Journal of Nursing Studies. 2009;46(2):189-96.

13. Thomas R, Worrall G, Elgar F, Knight J. Can they keep going on their own? A four-year randomized trial of functional assessments of community residents. Canadian Journal on Aging. 2007;26(4):379-89.

14. Callahan CM, Boustani MA, Unverzagt FW, Austrom MG, Damush TM, Perkins AJ, et al. Effectiveness of collaborative care for older adults with Alzheimer disease in primary care: A randomized controlled trial. Journal of the American Medical Association. 2006;295(18):2148-57.

15. Challis D, Clarkson P, Williamson J, Hughes J, Venables D, Burns A, et al. The value of specialist clinical assessment of older people prior to entry to care homes. Age & Ageing. 2004;33(1):25-34.

16. Crotty M, Giles LC, Halbert J, Harding J, Miller M. Home versus day rehabilitation: a randomised controlled trial. Age & Ageing. 2008;37(6):628-33.

17. Forster A, Young J, Green J, Patterson C, Wanklyn P, Smith J, et al. Structured re-assessment system at 6 months after a disabling stroke: a randomised controlled trial with resource use and cost study. Age & Ageing. 2009;38(5):576-83.

18. Montgomery RJ, Borgatta EF. The effects of alternative support strategies on family caregiving. The Gerontologist. 1989;29(4):457-64.

19. Montgomery RJ, Gonyea JG, Hooyman NR. Caregiving and the experience of subjective and objective burden. Family Relations: An Interdisciplinary Journal of Applied Family Studies. 1985;34(1):19-26.

20. Oktay JS, Volland PJ. Post-hospital support program for the frail elderly and their caregivers: A quasi-experimental evaluation. American Journal of Public Health. 1990;80(1):39-46.

21. Ryynanen OP, Nousiainen P, Soini EJO, Tuominen S. Efficacy of a multicomponent support programme for the caregivers of disabled persons. A randomised controlled study. Z Gerontol Geriatr. 2013;46(5):449-55.

22. Stewart M, Sangster JF, Ryan BL, Hoch JS, Cohen I, McWilliam CL, et al. Integrating physician services in the home: Evaluation of an innovative program. Canadian Family Physician. 2010;56(11):1166-74.

23. Tibaldi V, Aimonino N, Costamagna C, Obialero R, Ruatta C, Stasi MF, et al. Clinical outcomes in elderly demented patients and caregiver's stress: A 2-year follow-up study. Archives of Gerontology and Geriatrics. 2007;44:401-6.

24. Tourigny A, Durand P, Bonin L, Hebert R, Rochette L. Quasi-experimental study of the effectiveness of an integrated service delivery network for the frail elderly. Canadian Journal on Aging. 2004;23(3):231-46.

25. Vellas B, Hausner L, Frolich L, Cantet C, Gardette V, Reynish E, et al. Progression of Alzheimer disease in Europe: Data from the European ICTUS study. Current Alzheimer Research. 2012;9(8):902-12.

26. Vincent C, Reinharz D, Deaudelin I, Garceau M, Talbot LR. Public telesurveillance service for frail elderly living at home, outcomes and cost evolution: A quasi experimental design with two follow-ups. Health and Quality of Life Outcomes. 2006;4.

27. Whitlatch CJ, Zarit SH, Goodwin PE, von Eye A. Influence of the success of psychoeducational interventions on the course of family care. Clinical Gerontologist: The Journal of Aging and Mental Health. 1995;16(1):17-30.

28. Lim WK, Lambert SF, Gray LC. Effectiveness of case management and post-acute services in older people after hospital discharge. Medical Journal of Australia. 2003;178(6):262-6.

29. Lindpaintner LS, Gasser JT, Schramm MS, Cina-Tschumi B, Muller B, Beer JH. Discharge intervention pilot improves satisfaction for patients and professionals. European Journal of Internal Medicine. 2013;24(8):756-62.

30. Kalra L, Evans A, Perez I, Melbourn A, Patel A, Knapp M, et al. Training carers of stroke patients: randomised controlled trial. Bmj. 2004;328(7448):1099.

31. Mohide E, Pringle DM, Streiner DL, Gilbert J, Muir G, Tew M. A randomized trial of family caregiver support in the home management of dementia. Journal of the American Geriatrics Society. 1990;38(4):446-54.

32. Weinberger M, Gold DT, Divine GW, Cowper PA, Hodgson LG, Schreiner PJ, et al. Social service interventions for caregivers of patients with Dementia - Impact on health care utilization and expenditures Journal of the American Geriatrics Society. 1993;41(2):153-6.

33. Wilkinson PR, Wolfe CD, Warburton FG, Rudd AG, Howard RS, Ross-Russell RW, et al. A long-term follow-up of stroke patients. Stroke. 1997;28(3):507-12.

34. Hughes SL, Weaver FM, Giobbie-Hurder A, Manheim L, Henderson W, Kubal JD, et al. Effectiveness of team-managed home-based primary care - A randomized multicenter trial. JAMA-J Am Med Assoc. 2000;284(22):2877-85.

35. Jette AM, Tennstedt S, Crawford S. How does formal and informal community care affect nursing home use? J Gerontol B Psychol Sci Soc Sci. 1995;50(1):S4-S12.

36. Tsuji I, Whalen S, Finucane TE. Predictors of nursing home placement in community based long term care. Journal of the American Geriatrics Society. 1995;43(7):761-6.

37. Gilley DW, McCann JJ, Bienias JL, Evans DA. Caregiver psychological adjustment and institutionalization of persons with Alzheimer's disease. Journal of Aging & Health. 2005;17(2):172-89.

38. Lawton MP, Moss M, Kleban MH, Glicksman A, Rovine M. A two-factor model of caregiving appraisal and psychological well-being. Journal of gerontology. 1991;46(4):P181-9.

39. Aneshensel CS, Pearlin LI, Schuler RH. Stress, role captivity and the cessation of caregiving. J Health Soc Behav. 1993;34(1):54-70.

40. Pearlin LI, Mullan JT, Semple SJ, Skaff MM. Caregiving and the Stress Process: An Overview of Concepts and Their Measures. The Gerontologist. 1990;30(5):583-94.

41. McCann JJ, Hebert LE, Li Y, Wolinsky FD, Gilley DW, Aggarwal NT, et al. The effect of adult day care services on time to nursing home placement in older adults with Alzheimer's disease. Gerontologist. 2005;45(6):754-63.

42. Kurasawa S, Yoshimasu K, Washio M, Fukumoto J, Takemura S, Yokoi K, et al. Factors influencing caregivers' burden among family caregivers and institutionalization of in-home elderly people cared for by family caregivers. Environ. 2012;17(6):474-83.
