# Supplementary material for: Systematic Review and Meta-Analysis of the Impact of Carer Stress on Subsequent Institutionalisation of Community-Dwelling Older People
Source: PLoS One. 2015 Jun 2;10(6):e0128213. doi: 10.1371/journal.pone.0128213 (PMC4452721; doi:10.1371/journal.pone.0128213)
Supplement: S4 Appendix — (DOCX) [file pone.0128213.s004.docx]

**S4 Appendix: Characteristics of included studies**

| **Table 3 Characteristics of included studies** | | | | | | | | |
| --- | --- | --- | --- | --- | --- | --- | --- | --- |
| **Author; country; year of publication** | **Research design** | **Study population; care recipient % men; care recipient age mean (SD)** | **Sample size: baseline; follow-up** | **Exposure measure** | **Outcome measure** | **Quality score** | **Statistical results** | **Adjusted or unadjusted** |
| Bakker et al.[[1](#_ENREF_1)]; the Netherlands; 2013 | 2 year cohort | Dementia dyads. 55%.  YoD: 61.3 (SD=5.4); LoD: 80.9 (SD=5.8). | 328; 328 | Neuropsychiatric Inventory Distress scale (NPI-D). | Institutionalisation | 73% | Cox proportional Hazard Model - Caregiver distress (NPI-D): Hazard Ratio= 1.011; CI 0.975 - 1.048; p=.559. | Adjusted for young vs late onset dementia; carer age; carer sex; spousal carer; advanced dementia; diagnosis (Alzheimer’s dementia, front temporal dementia, mixed/vascular dementia, other dementias); neuropsychiatric symptoms (hyperactivity, psychosis, mood, apathy); caregiver distress (NPI-D); caregiver sense of competence (SSCQ); time from symptom onset at inclusion; interaction group with dementia severity; interaction group with apathy score. |
| Banerjee et al.[[2](#_ENREF_2)]; UK; 2003 | 1 year cohort | Dementia dyads. % men not provided. Mean (SD) age not provided. | 100; 69 | Zarit Burden Interview. | Institutionalisation | 30% | Logistic regression: carer burden (ZBI) Odds Ratio=1.02; CI 0.96-1.08; p=0.542. | Adjusted for age of person with dementia (pwd); female sex of pwd; female sex of carer; carer mental health; carer quality of life - psychological; person with dementia behaviour problems; carer burden score; person with dementia ADAS-cognitive score; spouse carer; co-resident carer. |
| Bannister et al.[[3](#_ENREF_3)]; UK; 1998 | 1 year cohort | Dementia dyads. 27% . 79.8 (SD not provided) | 124; 116 | Carer depression assessed using the GMS schedule [[4](#_ENREF_4)]. | Residential or nursing home care | 48% | Carer RDC depression: admitted 5 (20%); not admitted 26 (29%). | Unadjusted |
| Bond et al.[[5](#_ENREF_5)]; Australia; 2002 | 2 year cohort | Dementia dyads. % men continued receiving care: 50%; discontinued receiving care 64%. Continued receiving care age: 73.1 (SD 6.9); Discontinued receiving care age 75.2 (SD 6.5). | 163; 158 | Geriatric Depression Scale. | Yielding care to permanent supported accommodation | 50% | Carer geriatric depression scale score was significantly different in those continuing to care ( M=8.8; SD=6.2) compared to those yielding to care (M=11.2; SD=6.4; p<.05). | Unadjusted |
| Brodaty et al.[[6](#_ENREF_6)]; Australia; 1993 | 5 year controlled intervention study | Dementia dyads. 51%. 70.2 (SD not provided) | 91; 91 | Stress measured with General Health Questionnaire. | Actual nursing home placement | 40% | Impairment on GHQ (carer stress) was significant predictor of placement (p<.05). Those institutionalised n=69; not institutionalised n=22. | Adjusted for type of dementia; age; gender; severity of dementia; deterioration of dementia; degree of neuroticism of carer; degree of carer satisfaction with personal contacts; degree of satisfaction with telephone contacts. |
| Brown et al.[[7](#_ENREF_7)]; United States; 1990 | 1 year cohort | Subjects of the geriatric assessment programme of the University of Nebraska Medical Centre. 30%. 72 (SD 7.2) | 109; 109 | Zarit Burden Interview. | Nursing home placement | 55% | Significant difference in T-test in caregiver burden for individuals whose CR was admitted to a nursing home (n=27) compared to those where no placement took place (n=82; p<.01). | Unadjusted |
| Camden et al.[[8](#_ENREF_8)]; UK; 2011 | 1 year cohort | Dementia dyads. 85%. 83.1 (SD=8.0) | 111; 111 | Zarit Burden Interview. Carer anxiety with anxiety subscale of HADS. | Living in 24-hr care | 83% | ZBI: Cox regression for admission to 24-hour care, burden score: Hazard Ratio=1.023; 95%CI=0.02-.069; p=0.255..  HADS: Cox regression for admission to 24-hour care, carer anxiety score: Hazard Ratio=0.964; 95% CI=0.85-1.09; p=0.553. | Adjusted for negative vs positive reason for caring; neutral vs positive reason; carer gender; carer age; care recipient gender; care recipient age; child of care recipient; NPI score; ADL score; MMSE score; burden score; carer anxiety score. |
| Chuang et al.[[9](#_ENREF_9)]; Taiwan; 2005 | 1 month cohort | Stroke patients. % men readmission: 25%; % men non-readmission: 75%. Readmission: 73.8 (SD=9.77); Non-readmission: 73.0 (SD=10.7) | 489; 489 | Carer burden index. | Readmission to hospital within 1 month | 85% | Stressed n=149; n not stressed=340; n stressed admitted=47; n not stressed admitted=102. Chi square for differences between burden groups =9.32; p<.01. | Unadjusted |
| Cohen et al.[[10](#_ENREF_10)]; Canada; 1993 | 18 month cohort | Dementia dyads. % men not provided. 77.46 (no SD given) | 196; 156 | Zarit Burden Interview. | Institutionalisation | 50% | Carers who placed their dependent at 18 months (n=100) reported higher levels of burden (M=42.92; SD=15.25) than those who did not (n=56; M=37.02; SD=14.32) [f (1,132) =4.37; p<.05]. | Unadjusted |
| Cohen-Mansfield and Wirtz.[[11](#_ENREF_11)]; United States; 2007 | 3 year cohort | Adult day care participants. 34%. 79.96 (SEM 8.00) | 201; 199 | Zarit Burden Interview. | Any nursing home admission | 65% | Bivariate analysis with cox regression: caregiver burden risk ratio =1.91; p=.001; CI 1.29-2.82. | Unadjusted |
| Colerick and George.[[12](#_ENREF_12)]; United States; 1986 | 1 year cohort | Alzheimer's Disease dyads. % men not provided. Home caregiving: 68.71 (SD not provided); Became institutionalised: 68.89 (SD not provided). | 510; 209 | Stress measured with Langer 22-item screening scale [[13](#_ENREF_13)]. | Institutionalisation | 45% | Stress symptoms for those who continued home caregiving: M=8.75; those who became institutionalised: M=9.41; P=<.05. | Unadjusted |
| Deimling et al.[[14](#_ENREF_14)]; United States; 1985 | 6 month cohort | Families living with and caring for an older relative. % men not provided. Mean (SD) age not provided. | 64; 64 | Stress effects with composite measured created for study. Zung depression scale. | Institutionalisation | 43% | Carer’s stress scores of those who institutionalised the care recipient (M=8.38) were not significantly different from those who did not (M=6.61; F=3.222;p=>.05). Carer’s depression scores of those who institutionalised the care recipient (M=49.01) were not significantly different from those who did not (M=48.32; F=0.076;p=>.05). | Unadjusted |
| Drame et al.[[15](#_ENREF_15)]; France; 2013 | 6 month cohort | Patients aged 75 and over hospitalised via the Emergency Department. 35%. 85 (SD=6) | 1047; 1047 | Zarit Burden Interview. | Nursing home was defined as any type of residential care establishment or long-term care hospital unit | 95% | Admitted n=184; n not admitted=863. Caregiver burden p<0.001. | Unadjusted |
| Fisher, Lieberman.[[16](#_ENREF_16)]; United States; 1999 | 2 year cohort | Alzheimer's Disease dyads. 46%. Non NHP: 76 (SD 7.6); NHP: 78 (SD 6.4). | 211; 164 | Carer strain 11-item scale developed by Niedereche and Fruge [[17](#_ENREF_17)]. | Nursing home placement | 50% | No significant difference was found between those who placed their care recipient in a nursing home (n=79; carer strain M=22.7; SD= 5.2) and those who didn't (n=85; carer M=22.1; SD= 5.6; t=0.78; p=.43). | Unadjusted |
| Gaugler et al.[[18](#_ENREF_18)]; United States; 2005 | 3 year cohort | Dementia dyads. 40%. 79 (SD=8.8). | 5831; 5831 | Zarit burden Interview. | Long term nursing home placement | 60% | ZBI burden score admitted M=13.2; SD=6.36. Not admitted M=12.02; SD=6.34. | Unadjusted |
| Haupt, Kurz.[[19](#_ENREF_19)]; UK; 1993 | 1 year cohort | Alzheimer's Disease dyads. 25%. 73 (SD not provided) | 90; 66 | Items of the Zarit burden interview were used. | Nursing home placement | 40% | No significance was found between carer burden scores in those who did not institutionalise (M=2.8; SD=1.0) and those who did (M=3.3; SD 0.9; F=3.12) institutionalise their care recipient | Unadjusted |
| Hebert et al.[[20](#_ENREF_20)]; Canada; 2001 | 5 year cohort | Dementia dyads. % men not provided. Mean (SD) age not provided. | 326; 293 | Zarit burden Interview. Depression with CES-D. | Both NHP and Acute care admission longer than 3 months. | 63% | Severe carer burden was found to be significantly associated with a shorter time for institutionalisation controlling for ADL rating only HR=1.77; 95CI=1.28-2.44. Carer depression was found to be significantly associated with a shorter time for institutionalisation controlling for ADL rating only HR=1.04; 95 CI=1.01-1.07. | Adjusted for ADL rating only |
| Kodama et al.[[21](#_ENREF_21)]; Japan; 2009 | 2 year cohort | Caregivers of self-care-dependent elderly. 35%. Men: 77 (SD=7), Women: 82 (SD=8). | 1036; 465 (analysis from carers in ‘good’ relationship) | Depression with CES-D. | Institutionalisation | 50% | Carers in a good relationship whose care recipient was admitted to a nursing home (n=54) had a mean CESD score of 5.8 (SD=3.6). Carers who continued caring (n=411) had a mean CESD score of 5.4(SD=3.4). | Unadjusted |
| Kramer.[[22](#_ENREF_22)]; United States; 2000 | 1 year cohort | Dementia dyads. % men not provided. Mean (SD) age not provided. | 74; 57 | Burden with Memory and Behaviour Problem checklist. Depression with CES-D. | Institutionalisation | 48% | Significance difference between carers stress scores in those who did not institutionalise (M=18.98) and those who did (M=25.00; T=-2.23; p=<.05). No significance difference found between carers depression scores in those who did not institutionalise (M=13.65) and those who did (M=15.86; T=0.66) | Unadjusted |
| Kuzuya et al.[[23](#_ENREF_23)]; Japan; 2011 | 3 year cohort | Community dwelling elderly with some degree of physical or mental impairment. 36%. ZBI score 0-15:81 (SD=7.1); score 16-26:81(SD=7.7); score 27-39:81 (SD=7.8). Score 40-84:81(SD=8.5). | 1067; 935 | Zarit burden Interview. | Hospitalisation | 63% | Mean burden scores for those who continued home care was 27.26 (SD=17.12), while those whose CR was admitted to a nursing home was 30.46 (SD=16.9) | Unadjusted |
| Lieberman, Kramer.[[24](#_ENREF_24)]; United States; 1991 | 1 year cohort | Dementia dyads. 36%. 74 (SD=10.2). | 321; 321 | Caregiver stress assessed by the presence or absence of the number of reported family problems associated with caregiving (e.g. financial, psychological and social). | Institutionalisation | 43% | Stress of carers whose care recipient was institutionalised (M=1.9; SD=1.4) was significantly higher than those who remained in the community (M=1.4; SD=1.2; beta=.18; t=2.9; p=.00). | Unadjusted |
| Molloy et al.[[25](#_ENREF_25)]; Canada; 1999 | 3 year cohort | Dementia dyads. 43%. 72 (SD not provided). | 30; 30 | Zarit burden Interview. | Institutionalisation | 60% | Burden score was not predictive of institutionalisation (n admitted=10; n not admitted=20); p=.625). | Unadjusted |
| Nygaard HA.[[26](#_ENREF_26)]; United States; 1991 | 1 year cohort | Dementia dyads. % men not provided.  Admitted: 84 (71-92). Not admitted: 82 (62-93). | 46; 46 | Strain measured on a scale developed for study. | Admitted to a nursing home | 50% | No significance found in strain between those admitted (n=32) and those not admitted (n=14); p=0.7 | Unadjusted |
| Ohwaki et al.[[27](#_ENREF_27)]; Japan; 2009 | 1 year cohort | Elderly people living in the community eligible for care under long-term care insurance. 39%. Mean not provided. 30% aged 65-74; 36% aged 75-84; 34% aged 85. | 244; 244 | Zarit burden Interview. | Placement was to both nursing home and hospital admission. | 58% | There was no significant difference found in burden levels between those who continued home care (n=200) and those who did not (n=44; p=0.91). | Unadjusted |
| Oura et al.[[28](#_ENREF_28)]; Japan; 2006 | 5 year cohort | Frail elderly receiving in-home care. 43%. 81 (SD=8.5). | 122; 113 | Carer depression with CES-D. | Institutionalisation | 35% | Carer depression HR=1.32; 95CI=0.49-3.56. | Adjust for CR characteristics: gender; age; dementia; dementia with behavioural disturbance; Carer characteristics: Gender; age; depression; consulted with a doctor about their own health; spouse; daughter-in-law; Care setting: family member helped with caregiving; able to go out without accompanying the elderly. |
| Philp et al.[[29](#_ENREF_29)]; UK; 1997 | 2 year cohort | Dementia dyads. 2%. 80.7 (66-97). | People with Dementia: 114; 103 | Carer stress assessed by asking the carer to rate their overall level of stress using a 3-category response ('a lot', 'some', and 'hardly and/none'). | Institutionalisation was defined entry to residential or nursing home on a permanent basis; or a long-stay bed in a geriatric or psychiatric hospital | 48% | Carer stress was significantly associated with institutionalisation (to both hospital and NH) p=0.020 (n stressed=40; n not stressed=63; n stressed admitted=24; n not stressed admitted=25). | Unadjusted |
| Pot et al.[[30](#_ENREF_30)]; the Netherlands; 2001 | 1 year cohort | Dementia dyads. 30%. 78 (SD not provided). | 175; 138 | Perceived stress measured with the `Self-Perceived Pressure from Informal Care' questionnaire (SPPIC) [[31](#_ENREF_31)]. Psychological distress with the General Health Questionnaire (GHQ). | Admission to either a residential home or a nursing home. | 65% | There was a significant difference in stress between those whose care recipient remained at home (M=4.53; SD not provided) and those whose care recipient was institutionalised (M=5.52; SD not provided; t= -2.24; p=<0.05). No significance was found between psychological distress of carers whose care recipient remained at home (M=3.90, SD not provided) and those whose care recipient was institutionalised (M=4.61; SD not provided); t= -1.11; p=>.05. | Unadjusted |
| Pruchno et al.[[32](#_ENREF_32)]; United States; 1990 | 1 year cohort | Alzheimer's Disease dyads. % men not provided. Mean (SD) age not provided. | 315; 220 | Burden with a measure derived for the study. Depression with the CES-D. | Institutionalisation | 43% | Significant differences in burden were found between carers whose CR remained in the community (M=32.75) and those who were institutionalised (M=35.85; t=2.86; p=<.01). Significant differences in depression were found between carers whose CR remained in the community (M=14.93) and those who were institutionalised (M=20.16; t=3.16; p=<.01). | Unadjusted |
| Rongve et al.[[33](#_ENREF_33)]; Norway; 2013 | 6 year cohort | Dementia dyads. 45%. 76 (SD=7.7). | 189; 165 | Carer distress with the Relative's Stress Score [[34](#_ENREF_34)]. | Permanent or alternating residing in nursing homes or home for the elderly | 80% | Higher carer distress for those to a nursing home (M=16.3 SD=10.9) than those not admitted (M=14.7; SD=9.7). | Unadjusted |
| Schulz et al.[[35](#_ENREF_35)]; United States; 2004 | 18 month RCT | Dementia dyads. 44%. 79 (SD=8.2). | 1222; 1177 | Carer stress assessed with the Revised Memory and Behaviour Problem Checklist (RMBPC) [[36](#_ENREF_36)]. | Institutionalisation | 63% | Caregivers reporting greater burden were more likely to institutionalise their relative HR=1.02; 95CI=1.01-1.03; P=<.001. | Adjusted for carer race; RMBPC; MMSE; and positive aspects of caregiving. |
| Schwarz, Elman.[[37](#_ENREF_37)]; United States; 2003 | 3 month cohort | Patients with Heart Failure and their carers. 52%. 77 (SD =6.1). | 156; 128 | Perceived stress measured with the Perceived Stress Scale [[38](#_ENREF_38)].  Depression measured with the CES-D. | Hospital readmission | 60% | No significant differences were found in stress scores between those re-admitted to hospital (M=16.18; SD=8.3) and not those re-admitted (M=16.75; SD=9.2; p=.55). No significant differences were found in depression scores between those re-admitted to hospital (M=10.87; SD=8.5) and not re-admitted (M=11.86; SD=9.1; p=.86). | Unadjusted |
| Shugarman et al.[[39](#_ENREF_39)]; United States; 2002 | 3 month cohort | Home Care Support participants. 30%. 75 (SD=11.6). | 527; 527 | Burden measured with items from the MDS-HC [[40](#_ENREF_40)]. | Hospital readmission | 60% | Burdened carers= 106; non-burden carers=421; burdened carers whose recipient was admitted=17; non-burdened carers whose recipient was admitted=67. | Unadjusted |
| Soto et al.[[41](#_ENREF_41)]; France; 2006 | 1 year cohort | Alzheimer's Disease dyads. % men not provided. Mean (SD) age not provided. | 455; 455 | Zarit burden Interview. | Institutionalisation | 58% | The OR for those with moderate Zarit score was OR=1.21; 95 CI=0.46-3.16; p=0.693. | Adjusted for MMSE, living arrangements and Zarit score |
| Spillman, Long.[[42](#_ENREF_42)]; United States; 2009 | 2 year cohort | Older people aged 65+ with chronic disabilities with an established caregiving arrangement. 32%. 80 (SD not provided). | 1006; 1006 | Carer's reports of frequent recipient behaviour problems and of experiencing strain from providing care along with the overall level of stress the caregiver reports experiencing from caregiving responsibilities. | Nursing home entry considered admissions to nursing homes for episodes of care that last at least 60 days | 48% | Stressed carers=191; non-stressed carers=815; stressed carers whose recipient was admitted=33; non-stressed carers whose recipient was admitted=98. | Unadjusted |
| Spruytte et al.[[43](#_ENREF_43)]; Belgium; 2001 | 9 month cohort | Dementia dyads. 31%. 82 (61-94). | 144; 109 | Self-Perceived Pressure from Informal Care [[31](#_ENREF_31)]. | Institutionalisation | 68% | For those admitted N=21; M=39.6; SD=13.4. For those not admitted N=88; M=43.1; SD=9.1. | Unadjusted |
| Stevens et al.[[44](#_ENREF_44)]; United States; 2004 | 2 year cohort | Dementia dyads. 29%. 74 (SD=8.2). | 215; 215 | Burden with Memory and Behaviour Problem Checklist. Depression with CES-D. | Nursing home placement | 60% | Carer stressfulness appraisal of memory and behaviour problems demonstrated significant influence on time to placement ( n admitted=59; n not admitted=156; p=.033). CESD did not demonstrate significant influence on time to placement( n admitted=59; n not admitted=156 p=.68). | Unadjusted |
| Strain et al.[[45](#_ENREF_45)]; Canada; 2003 | 5 year cohort | Older adults diagnosed with cognitive impairment-not dementia or dementia. 49%. 81 (SD not provided). | 123; 123 | Zarit burden Interview. | Institutionalisation | 65% | Carer burden in high risk: OR=1.03 95CI=0.93-1.14. Medium risk, OR=1.03 95CI=0.96-1.10. Low-risk: OR=1.04 95CI=0.97-1.11. | Unadjusted |
| Thorpe et al.[[46](#_ENREF_46)]; United States; 2010 | 1 year cohort | Dementia dyads. % men not provided. 76 (SD=5.3). | 1186; 1186 | Distress with CES-D. | Hospitalisation | 68% | Odds Ratio for CES-D=1.01 95CIs=0.98-1.04. | Unadjusted |
| Voisin et al.[[47](#_ENREF_47)]; France; 2010 | 2 year cohort | Dementia dyads. 30%. 78 (SD=6.82). | 686; 686 | Zarit Burden Interview. | Hospitalisation | 58% | Carer burden score RR=1.016; 95CI=1.008-1.025; p=.0002 | Unadjusted |
| Washio et al.[[48](#_ENREF_48)]; Japan; 2002 | 6 month cohort | Caregivers and the disabled elderly. 40%. 83 (SD=8.7). | 48; 48 | Depression with CES-D. | Admission to long term care institution | 50% | Stressed n=22; n not stressed=26; n stressed admitted=4; n not stressed admitted=3. | Unadjusted |
| Whitlatch et al.[[49](#_ENREF_49)]; United States; 1999 | 2 year cohort | Alzheimer's Disease dyads. % men not provided. 76 (49-97). | 926; 926 | Zarit burden Interview. Depression with CES-D. | Institutionalisation | 55% | Carer burden at baseline was significantly higher for those carers who placed the care recipient (M=19.2) than those who did not (18.0; t=6.92; p<.01). Carer depression at baseline was significantly higher for those carers who placed the care recipient (M=22.3) than those who did not (20.2; t=6.93; p<.01). | Unadjusted |
| Young et al.[[50](#_ENREF_50)]; United States; 1998 | 18 month cohort | Alzheimer's Disease dyads. 35%. 77 (SD not provided). | 575; 426 | Burden measured by 6 item scale developed by the author. | Institutionalisation | 50% | Mean burden scores for those who continued home care (n=261) was 18.8, while those whose CR was admitted to a nursing home (n=165) was 21.7; p=<.01. | Unadjusted |
| Zarit, Todd, Zarit.[[51](#_ENREF_51)]; United States; 1986 | 2 year cohort | Dementia dyads. % men not provided. Husbands with dementia 69 (SD=7.22); Wives with dementia (71 (SD=7.12). | 64; 43 | Zarit burden Interview. | Institutionalisation | 48% | Initial burden scores were significantly higher for those carer who placed their spouse in a nursing home (M=43.54) than those who did not (M=33.59; t=2.23; p<.05). | Unadjusted |
| Gruneir et al.[[52](#_ENREF_52)]; Canada; 2013 | 1 year cohort | Community dwelling dyads. % men not provided. Mean (SD) age not provided. | 71303; 71303 | Carer distress with measure from InterRAI HC assessment [[40](#_ENREF_40)]. | Admission to acute care | 88% | Stressed n=8,720; n not stressed=62,583; n stressed admitted=3,628; n stressed not admitted=22,572. | Unadjusted |
| Baumgarten et al.[[53](#_ENREF_53)]; Canada; 1994 | 1 year cohort | Dementia dyads. 46%. 79 (SD not provided). | 86; 86 | Depression with the CES-D. | Institutionalisation | 53% | Mean CES-D scores for those whose care recipient was institutionalised =15.8 (SD not provided) and those not institutionalise =15.5 (SD not provided). Admitted n=30; n not admitted=56; n stressed admitted=13; n not stressed admitted=17. | Unadjusted |
| Mittelman et al.[[54](#_ENREF_54)]; United States; 2006 | 18 year RCT | Alzheimer's Disease dyads. % men not provided. 74 (SD=8.38). | 406; 406 | Zarit burden Interview. Depression with geriatric depression scale. | Nursing home placement | 53% | The carer burden HR=1.009; 95CI=0.996-1.021; p=0.1759. The carer depression HR=1.016; 95CI=0.988-1.044; p=0.2791 | Adjusted for group (intervention vs control); carer gender; carer age; patient age; patient income; year of study entry; global deterioration scale; carer physical health; patient physical health; satisfaction with support network; frequency of memory and behaviour problems; reaction to memory and behaviour problems; depressive symptoms and carer burden. |
| Nobili et al.[[55](#_ENREF_55)]; Italy; 2004 | 12 month RCT | Dementia dyads. % men in control=41%; intervention=40%. Control=75 (SD=10); Intervention=74 (SD=9). | 69; 55 | carer stress measured with relative stress scale (RSS) [[34](#_ENREF_34)]. | Institutionalisation | 65% | The mean stress score of those whose care recipient was institutionalised M= 36 (SD=8), for those whose care recipient was not institutionalised M=26 (SD=11). | Unadjusted |
| Spijker et al.[[56](#_ENREF_56)]; Netherlands; 2011 | 12 month RCT | Suspected Dementia dyads. %men in control=36%; % men intervention=30%. Control=80 (SD=6.4); Intervention=80 (SD=7.1). | 295; 262 | Carer depression CES-D. | Institutionalisation in long-term care facilities. | 80% | Log regression, carer depression OR=1.05; 95% CI=1.01-1.09; p=0.01. | Adjusted for group assignment (intervention vs control); gender; living arrangement; severity of dementia; sense of competence; behavioural problems; carer depressive symptoms; group assignment x sense of competence; gender x living arrangement |
| Joling et al.[[57](#_ENREF_57)]; The Netherlands; 2012 | 18 month RCT | Dementia dyads. 67%. 77 (SD=8.3). | Control group: 96; 95 | Neuropsychiatric Inventory Questionnaire (NPI-Q) distress score. Depression measured with CES-D. | Institutionalisation | 78% | Admitted n=18; n not admitted=77. Carer distress admitted M=14.7; SD=7.6; not admitted M=12.1; SD=9.6. Carer depression admitted M=12.8 SD=5.5; not admitted M=10.3; SD=7.4. | Unadjusted |
| Phung et al.[[58](#_ENREF_58)]; Denmark; 2013 | 3 year RCT | Alzheimer's Disease dyads. 45%. 76 (SD=6.6). | Control group: 167; 167 | Depression measured with Geriatric depression scale. | Nursing home placement | 80% | Admitted n=48; M=4.38; SD=5.3. Not admitted n=119; M=4.84; SD=4.9. | Unadjusted |
| Wai Tong, Lee.[[59](#_ENREF_59)]; China; 2011 | 18 month RCT | Dementia dyads. 56%. 69 (SD=6.8). | Control group: 46; 45 | Family Caregiving Burden Inventory (FCBI). | Institutionalisation | 93% | Stressed n=25; n not stressed=13; n stressed admitted=18; n not stressed admitted=9. | Unadjusted |
| Wang, Chien.[[60](#_ENREF_60)]; China; 2011 | 7 month RCT | Dementia dyads. 54%. 68 (SD=7.9). | Control group: 40; 40 | Family Caregiving Burden Inventory (FCBI). | Institutionalisation | 80% | Burdened carers= 20; non-burden carers=19; burdened carers whose recipient was admitted=13; non-burdened carers whose recipient was admitted=8. | Unadjusted |
| Droes et al.[[61](#_ENREF_61)]; The Netherlands; 2006 | 18 month non randomised pre and post-test design | Dementia dyads, % men not provided. Mean (SD) age not provided. | Control group: 34; 18 | General Health Questionnaire (GHQ-28). | Nursing home placement | 50% | Admitted n= 5; M=34.6; SD=17.97; not admitted n=13; M=29; SD=17.9. | Unadjusted |
| Kurz et al.[[62](#_ENREF_62)]; Austria, Switzerland and Germany; 2010 | 15 month RCT | Alzheimer's Disease dyads. 32%. 76 (SD not provided) | Control group: 132; 106 | Montgomery-Asberg Depression Scale. | Permanent nursing home placement | 73% | Admitted n= 12; M=11.00; SD=9.24; not admitted n=94; M=11.15; SD=8.87.. | Unadjusted |

# References

1. Bakker C, de Vugt ME, van Vliet D, Verhey FR, Pijnenburg YA, Vernooij-Dassen MJ, et al. Predictors of the time to institutionalization in young- versus late-onset dementia: results from the Needs in Young Onset Dementia (NeedYD) study. Journal of the American Medical Directors Association. 2013;14(4):248-53.

2. Banerjee S, Murray J, Foley B, Atkins L, Schneider J, Mann A. Predictors of institutionalisation in people with dementia. J Neurol Neurosurg Psychiatry. 2003;74(9):1315-6.

3. Bannister C, Ballard C, Lana M, Fairbairn A, Wilcock G. Placement of dementia sufferers in residential and nursing home care. Age and Ageing. 1998;27(2):189-93.

4. Copeland JR, Kelleher MJ, Kellett JM, Gourlay AJ, Gurland BJ, Fleiss JL, et al. A semi-structured clinical interview for the assessment of diagnosis and mental state in the elderly: the Geriatric Mental State Schedule. I. Development and reliability. Psychol Med. 1976;6(3):439-49.

5. Bond MJ, Clark MS. Predictors of the decision to yield care of a person with dementia. Australasian Journal on Ageing. 2002;21(2):86-91.

6. Brodaty H, McGilchrist C, Harris L, Peters KE. Time until institutionalization and death in patients with dementia. Role of caregiver training and risk factors. Archives Of Neurology. 1993;50(6):643-50.

7. Brown LJ, Potter JF, Foster BG. Caregiver burden should be evaluated during geriatric assessment. Journal of the American Geriatrics Society. 1990;38(4):455-60.

8. Camden A, Livingston G, Cooper C. Reasons why family members become carers and the outcome for the person with dementia: results from the CARD study. International Psychogeriatrics. 2011;23(9):1442-50.

9. Chuang KY, Wu SC, Ma AH, Chen YH, Wu CL. Identifying factors associated with hospital readmissions among stroke patients in Taipei. J Nurs Res. 2005;13(2):117-28.

10. Cohen CA, Gold DP, Shulman KI, Wortley JT, McDonald G, Wargon M. Factors determining the decision to institutionalize dementing individuals: A prospective study. The Gerontologist. 1993;33(6):714-20.

11. Cohen-Mansfield J, Wirtz PW. Characteristics of adult day care participants who enter a nursing home. Psychol Aging. 2007;22(2):354-60.

12. Colerick EJ, George LK. Predictors of institutionalization among caregivers of patients with Alzheimer's disease. Journal of the American Geriatrics Society. 1986;34(7):493-8.

13. Langner TS. A twenty-two item screening score of psychiatric symptoms indicating impairment. Journal of health and human behavior. 1962;3:269-76.

14. Deimling GT, Poulshock S. The transition from family in-home care to institutional care: Focus on health and attitudinal issues as predisposing factors. Research on Aging. 1985;7(4):563-76.

15. Drame M, Mahmoudi R, Jolly D, Rapin A, Morrone I, Boyer FC, et al. Social support and six-month outcome among elderly patients hospitalised via emergency department: The SAFES Cohort Study. European Geriatric Medicine. 2013;4(3):161-6.

16. Fisher L, Lieberman MA. A longitudinal study of predictors of nursing home placement for patients with dementia: the contribution of family characteristics. The Gerontologist. 1999;39(6):677-86.

17. Niederehe G, Fruge E. Dementia and family dynamics: clinical research issues. J Geriatr Psychiatry. 1984;17(1):21-60.

18. Gaugler JE, Kane RL, Kane RA, Newcomer R. Unmet care needs and key outcomes in dementia. Journal of the American Geriatrics Society. 2005;53(12):2098-105.

19. Haupt M, Kurz A. Predictors of nursing home placement in patients with Alzheimer's Disease. International Journal of Geriatric Psychiatry. 1993;8(9):741-6.

20. Hebert R, Dubois M-F, Wolfson C, Chambers L, Cohen C. Factors associated with long-term institutionalization of older people with dementia: Data from the Canadian Study of Health and Aging. The Journals of Gerontology: Series A: Biological Sciences and Medical Sciences. 2001;56A(11):M693-M9.

21. Kodama H, Izumo Y, Takahashi R, Suda Y, Kudo H, Kudo H, et al. Family relationships of self-care-dependent older people and institutionalized rate to nursing homes. Geriatrics and Gerontology International. 2009;9(3):320-5.

22. Kramer BJ. Husbands caring for wives with dementia: A longitudinal study of continuity and change. Health & Social Work. 2000;25(2):97-107.

23. Kuzuya M, Enoki H, Hasegawa J, Izawa S, Hirakawa Y, Shimokata H, et al. Impact of caregiver burden on adverse health outcomes in community-dwelling dependent older care recipients. American Journal of Geriatric Psychiatry. 2011;19(4):382-91.

24. Lieberman MA, Kramer JH. Factors affecting decisions to institutionalize demented elderly. The Gerontologist. 1991;31(3):371-4.

25. Molloy DW, Bedard M, Pedlar D, Lever JA. Institutionalization in cognitively-impaired older individuals: a longitudinal study. Clinical Gerontologist. 1999;20(2):3-22.

26. Nygaard HA. Who cares for the caregiver? Factors exerting influence on nursing home admissions of demented elderly. Scandinavian Journal of Caring Sciences. 1991;5(3):157-62.

27. Ohwaki K, Hashimoto H, Sato M, Tamiya N, Yano E. Predictors of continuity in home care for the elderly under public long-term care insurance in Japan. Aging Clinical and Experimental Research. 2009;21(4-5):323-8.

28. Oura A, Washio M, Wada J, Arai Y, Mori M. Factors related to institutionalization among the frail elderly with home-visiting nursing service in Japan. Gerontology. 2006;52(1):66-8.

29. Philp I, McKee K, Armstrong G, Ballinger B, Gilhooly M, Gordon D, et al. Institutionalization risk amongst people with dementia supported by family carers in a Scottish city. Aging & Mental Health. 1997;1(4):339-45.

30. Pot AM, Deeg DJH, Knipscheer CPM. Institutionalization of demented elderly: The role of caregiver characteristics. International Journal of Geriatric Psychiatry. 2001;16(3):273-80.

31. Pot AM, van Dyck R, Deeg DJ. [Perceived stress caused by informal caregiving. Construction of a scale]. Tijdschr Gerontol Geriatr. 1995;26(5):214-9.

32. Pruchno RA, Michaels JE, Potashnik SL. Predictors of Institutionalization among Alzheimer's Disease victims with caregiving spouses J Gerontol. 1990;45(6):S259-S66.

33. Rongve A, Vossius C, Nore S, Testad I, Aarsland D. Time until nursing home admission in people with mild dementia: comparison of dementia with Lewy bodies and Alzheimer's dementia. International journal of geriatric psychiatry. 2014;29(4):392-8.

34. Greene JG, Smith R, Gardiner M, Timbury GC. Measuring behavioural disturbance of elderly demented patients in the community and its effects on relatives: a factor analytic study. Age and ageing. 1982;11(2):121-6.

35. Schulz R, Belle SH, Czaja SJ, McGinnis KA, Stevens A, Zhang S. Long-term care placement of dementia patients and caregiver health and well-being. Jama. 2004;292(8):961-7.

36. Teri L, Truax P, Logsdon R, Uomoto J, Zarit S, Vitaliano PP. Assessment of behavioral problems in dementia: the revised memory and behavior problems checklist. Psychology and aging. 1992;7(4):622-31.

37. Schwarz KA, Elman CS. Identification of factors predictive of hospital readmissions for patients with heart failure. Heart Lung. 2003;32(2):88-99.

38. Cohen S, Kamarck T, Mermelstein R. A global measure of perceived stress. Journal of health and social behavior. 1983;24(4):385-96.

39. Shugarman LR, Buttar A, Fries BE, Moore T, Blaum CS. Caregiver attitudes and hospitalization risk in michigan residents receiving home- and community-based care. Journal of the American Geriatrics Society. 2002;50(6):1079-85.

40. Morris JN, Nonemaker S, Murphy K, Hawes C, Fries BE, Mor V, et al. A commitment to change: revision of HCFA's RAI. Journal of the American Geriatrics Society. 1997;45(8):1011-6.

41. Soto ME, Andrieu S, Gillette-Guyonnet S, Cantet C, Nourhashemi F, Vellas B. Risk factors for functional decline and institutionalisation among community-dwelling older adults with mild to severe Alzheimer's disease: one year of follow-up. Age & Ageing. 2006;35(3):308-10.

42. Spillman BC, Long SK. Does High Caregiver Stress Predict Nursing Home Entry? Inquiry-J Health Care Organ Provis Financ. 2009;46(2):140-61.

43. Spruytte N, Van Audenhove C, Lammertyn F. Predictors of institutionalization of cognitively-impaired elderly cared for by their relatives. International Journal of Geriatric Psychiatry. 2001;16(12):1119-28.

44. Stevens A, Owen J, Roth D, Clay O, Bartolucci A, Haley W. Predictors of time to nursing home placement in White and African American individuals with dementia. Journal of Aging & Health. 2004;16(3):375-97.

45. Strain LA, Blandford AA, Mitchell LA, Hawranik PG. Cognitively impaired older adults: risk profiles for institutionalization. International Psychogeriatrics. 2003;15(4):351-66.

46. Thorpe JM, Van Houtven CH, Sleath BL, Thorpe CT. Rural-urban differences in preventable hospitalizations among community-dwelling veterans with dementia. J Rural Health. 2010;26(2):146-55.

47. Voisin T, Andrieu S, Cantet C, Vellas B. Predictive factors of hospitalizations in Alzheimer's disease: A two-year prospective study in 686 patients of the REAL.FR study. Journal of Nutrition, Health and Aging. 2010;14(4):288-91.

48. Washio M, Wada J-i, Tokunaga S, Arai Y, Mori M. Long-Term Care Insurance for Elderly and Depression among Caregivers of the Frail Elderly in Urban Japan: A Follow-Up Study. International Medical Journal. 2002;9(4):251-5.

49. Whitlatch CJ, Feinberg LF, Stevens EJ. Predictors of institutionalization for persons with Alzheimer's disease and the impact on family caregivers. Journal of Mental Health and Aging. 1999;5(3):275-88.

50. Young RF, Kosloski K, Montgomery RJ. Psychosocial factors in institutionalization of Alzheimer's patients. Journal of Clinical Geropsychology. 1998;4(3):241-51.

51. Zarit SH, Todd PA, Zarit JM. Subjective burden of husbands and wives as caregivers: A longitudinal study. The Gerontologist. 1986;26(3):260-6.

52. Gruneir A, Forrester J, Camacho X, Gill SS, Bronskill SE. Gender differences in home care clients and admission to long-term care in Ontario, Canada: a population-based retrospective cohort study. BMC geriatr. 2013;13:12.

53. Baumgarten M, Hanley JA, Infante-Rivard C, Battista RN, Becker R, Gauthier S. Health of family members caring for elderly persons with dementia. A longitudinal study. Annals of Internal Medicine. 1994;120(2):126-32.

54. Mittelman MS, Haley WE, Clay OJ, Roth DL. Improving caregiver well-being delays nursing home placement of patients with Alzheimer disease. Neurology. 2006;67(9):1592-9.

55. Nobili A, Riva E, Tettamanti M, Lucca U, Liscio M, Petrucci B, et al. The effect of a structured intervention on Caregivers of patients with dementia and problem behaviors - A randomized controlled pilot study. Alzheimer Dis Assoc Disord. 2004;18(2):75-82.

56. Spijker A, Wollersheim H, Teerenstra S, Graff M, Adang E, Verhey F, et al. Systematic care for caregivers of patients with dementia: a multicenter, cluster-randomized, controlled trial. American Journal of Geriatric Psychiatry. 2011;19(6):521-31.

57. Joling KJ, van Marwijk HWJ, van der Horst HE, Scheltens P, van de Ven PM, Appels BA, et al. Effectiveness of family meetings for family caregivers on delaying time to nursing home placement of dementia patients: A randomized trial. PLoS One. 2012;7(8).

58. Phung KTT, Waldorff FB, Buss DV, Eckermann A, Keiding N, Rishoj S, et al. A three-year follow-up on the efficacy of psychosocial interventions for patients with mild dementia and their caregivers: The multicentre, rater-blinded, randomised Danish Alzheimer Intervention Study (DAISY). BMJ Open. 2013;3(11).

59. Wai Tong C, Lee IM. Randomized controlled trial of a dementia care programme for families of home-resided older people with dementia. Journal of Advanced Nursing. 2011;67(4):774-87.

60. Wang L-Q, Chien W-T. Randomised controlled trial of a family-led mutual support programme for people with dementia. J Clin Nurs. 2011;20(15-16):2362-6.

61. Droes R, Meiland F, Schmitz M, Van Tilburg W. Effect of the meeting centres support program on informal carers of people with dementia: Results from a multi-centre study. Aging & Mental Health. 2006;10(2):112-24.

62. Kurz A, Wagenpfeil S, Hallauer J, Schneider-Schelte H, Jansen S. Evaluation of a brief educational program for dementia carers: the AENEAS study. International journal of geriatric psychiatry. 2010;25(8):861-9.
