# Supplementary material for: Systematic Review and Meta-Analysis of the Impact of Carer Stress on Subsequent Institutionalisation of Community-Dwelling Older People
Source: PLoS One. 2015 Jun 2;10(6):e0128213. doi: 10.1371/journal.pone.0128213 (PMC4452721; doi:10.1371/journal.pone.0128213)
Supplement: S5 Appendix — (DOCX) [file pone.0128213.s005.docx]

**S5 Appendix: PRISMA Checklist**

| **Section/topic** | **#** | **Checklist item** | | **Reported on page #** |
| --- | --- | --- | --- | --- |
| **TITLE** | | | |  |
| Title | 1 | Systematic review and meta-analysis of the impact of carer stress on subsequent institutionalisation of community dwelling older people | | 1 |
| **ABSTRACT** | | | |  |
| Structured summary | 2 | **Background:** In the caregiving literature there is a common belief that higher levels of carer stress is a critical determinant of premature ending of homecare. However, this contention has not been systematically analysed. We therefore systematically reviewed and meta-analysed the prospective association between various forms of carer stress and subsequent institutionalisation of community dwelling older people.  **Methods:** Systematic literature search of prospective studies measuring carer stress at baseline and institutionalisation at follow-up. Given substantial interchangeability in the measurement of carer stress, a wide number of exposure measures were included, namely: carer stress, burden, depression, distress, anxiety, burnout and strain. Institutionalisation included both acute and long-term care utilisation. The standardised mean difference between stressed and non-stressed carers was the primary measure of effect.  **Results:** The search yielded 6,963 articles. After exclusions, 54 datasets were analysed. The meta-analysis found that while carer stress has a significant effect on subsequent institutionalisation of care recipients the overall effect size was negligible (SMD=·05, 95% CI=·04 -·07; I^2^ = 79·2%; p=·000). The sensitivity analysis found that, whether analysing the association between carer stress, burden, distress or depression with either acute or long-term care, the effect size remains small to negligible. Estimates also reduce over time, with larger and better quality studies.    **Conclusion:** It would appear that over time, larger, better quality studies, adjusting for more factors have found less of an effect of carer stress on institutionalisation than was believed to be the case from initial studies. Taken together, the results suggest a need to re-examine the belief that carer stress could undermine the sustainability of homecare as according to the present results, carer stress does not appear to be a crucial factor in institutionalisation of care recipients. | | 1-2 |
| **INTRODUCTION** | | | |  |
| Rationale | 3 | In the caregiving literature there is a commonly held belief that, as a carer is a critical element of home care, if the level of stress on a carer becomes too great, the home care support provided by the carer may be seriously jeopardized.[[1](#_ENREF_1)] However, previous reviews on the predictors of institutionalisation have not taken into account the level of carer stress.[[2-6](#_ENREF_2)] Where carer stress was accounted for, it was found to consistently predict institutionalisation, though the analysis was confined to whether carer stress was significant and the direction of the effect, but not the size of the effect. [[7](#_ENREF_7)] Therefore, this systematic review and meta-analysis examines the longitudinal association between carer stress and institutional placement of the care recipient. | | 2-4 |
| Objectives | 4 | To examine the effect of carer stress on subsequent institutional placement of community dwelling older people. | | 5 |
| **METHODS** | | | |  |
| Protocol and registration | 5 | N/A | |  |
| Eligibility criteria | 6 | **Types of studies:** Control groups from intervention studies and, where data from control groups alone could not be obtained, combined intervention and control groups from randomised trials were also included. Studies were excluded if they were cross-sectional, retrospective or not written in English. Articles were not limited by year of publication.  **Types of participants:** *Care recipients*: Community-dwelling older people (aged 65 and over) with chronic care needs that are being cared for by an informal carer. *Carer:* Informal carer who takes primary responsibility of the care recipient. Articles with data on professional or paid carers were excluded.  **Types of exposures:** As different measures of psychological morbidity have been analysed in the prediction of institutional placement, the review included a wide number of exposure measures, namely carer stress, burden, depression, distress, anxiety, burnout and strain. Given the range of possible exposure variables they were considered in the following hierarchical manner: composite measure of burden and stress which have been tested for validity and reliability; composite measure of burden or stress yet to be tested for validity and reliability; composite measure of depression, distress, anxiety or strain which have been tested for validity and reliability. Where studies included more than one measure both were recorded and analysed separately in a sensitivity analysis. For the overall effect estimate the hierarchy was used to select the best estimate.  **Types of outcomes:** *Acute care utilisation:* Emergency Department visits and/or hospital admissions. *Long-term care utilisation*: Admission to a nursing home. These outcomes were not restricted in terms of the length of time the care recipient had been in an institution or the duration of follow-up. | | 5-6 |
| Information sources | 7 | CINAHL, Medline (OVID), PsycInfo, Web of Knowledge and EMBASE | | 6 |
| Search | 8 | **Search terms**: carer or caregiver; aged or elderly or Alzheimer or dementia; stress or burden or burnout or distress or anxiety or depression or strain; nursing home or long term care or long term care utilisation or care home or homes for the aged or institutionalisation or acute care or hospitalisation or hospital admission or hospital readmission or emergency department or accident and emergency. Appendix 1 provides an example of the search strategy for Medline (OVID).  **Example of MEDLINE (OVID) search strategy:**  #1 (carer$ or caregiver$).ab,kf,sh,ti.  #2 (dementia or alzheimer$ or elderly or aged).ab,kf,sh,ti.  #3 (stress or burden or burnout or distress or anxiety or depression or strain).ab,kf,sh,ti.  #4 (nursing home$ or Institutionali#ation or long term care or long term utilisation or care home or homes for the aged or acute care or hospitali#ation or (hospital admission or hospital readmission) or emergency department or emergency services department or (accident and emergency)).ab,ti,kf,sh.  #5 #1 AND #2 AND #3 AND #4 | | 6 |
| Study selection | 9 | The first reviewer screened all titles and abstracts of papers identified by the literature search (NAD). A second reviewer screened a random selection of fifteen percent of articles (AB). Disagreements were discussed with a third reviewer (FD). All studies identified as potentially relevant were retrieved and read in full to determine eligibility for inclusion. | | 7 |
| Data collection process | 10 | Data extraction was conducted by using a pre-defined data extraction template | | 7 |
| Data items | 11 | Extracted data included design characteristics; study population and country; sample size; length of follow up; sample selection; age and sex of participants; the exposure and outcome measures and results. Where there was insufficient data in the published paper authors were contacted to provide further information | | 7 |
| Risk of bias in individual studies | 12 | Assessment of the risk of bias was undertaken as part of the quality assessment of included studies. Quality assessment was conducted with the Crowe Critical Appraisal Tool (CCAT).[[8-10](#_ENREF_8)] The CCAT has undergone testing for reliability and validity and is considered a reliable means of appraising a wide range of research designs [[8](#_ENREF_8), [9](#_ENREF_9)]. | | 7 |
| Summary measures | 13 | The standardised mean difference (SMD) between stressed and non-stressed carers was the primary measure of effect. | | 7 |
| Synthesis of results | 14 | Effects were estimated in a random effects model for all included studies using the metaeff command in Stata (12·0).[[11](#_ENREF_11), [12](#_ENREF_12)] The I^2^ test was used to describe the percentage of total variation across studies that was due to heterogeneity rather than chance.[[13](#_ENREF_13)] | | 7 |
| Risk of bias across studies | 15 | Funnel plot asymmetry was assessed. The Egger’s test also allowed for assessment of publication bias or small study effects | | 8 |
| Additional analyses | 16 | Pre-planned sensitivity analysis included examination of estimates according to study size; the decade and regions in which studies were conducted; study quality as reviewed by the CCAT; use of adjusted or unadjusted estimates; dementia populations compared to non-dementia populations; different follow-up periods; and long term care in comparison to acute care utilisation; and the impact of different exposure measures. Given the methodological diversity of included studies significant heterogeneity was anticipated. Therefore a meta-regression was planned to understand the extent to which heterogeneity was related to the characteristics of the studies.[[14](#_ENREF_14)] | | 8-9 |
| **RESULTS** | | | |  |
| Study selection | 17 | | After duplicates were removed the search retrieved 4,701 articles; of which 4,582 were excluded (4,367 on review of abstract and a further 215 after full text assessment). A further 65 articles were omitted (27 repeat publications and in 38 additional studies adequate data was not available following contact with authors). Thus 54 studies were included in the analysis. Figure 1 presents a flow diagram of the search strategy. | 9 |
| Study characteristics | 18 | | Eighteen studies were conducted in Europe, twenty six in North America, eight studies in Asia and two were conducted in Australia (see appendix 3). In the majority of cases the research design adopted was a cohort study with study populations of caregivers and dementia care recipient dyads.  A number of studies referred to the same measure as a measure of burden, stress or distress. This interchangeability was seen both within and between studies. | 9-11 |
| Risk of bias within studies | 19 | | Assessment of the risk of bias was undertaken as part of the quality assessment of included studies. To examine the impact of study quality on effect estimates studies were grouped into quartiles. | 12 |
| Results of individual studies | 20 | | Summary data and effect estimates and confidence intervals for individual studies are displayed in figure 2. | 11-12 |
| Synthesis of results | 21 | | The meta-analysis found that, while carer stress has a significant effect on subsequent institutionalisation of care recipients, the overall effect size across the 54 studies was negligible (SMD=·05, 95% CI=·04 -·07). There was evidence of statistically significant heterogeneity (I^2^ =79·2%, p=<·001). | 11 |
| Risk of bias across studies | 22 | | There was evidence of funnel plot asymmetry (see Figure 3). Furthermore, the Egger’s bias coefficient (bias =1·45 P> \|t\| =<·001) strongly indicates the presence of asymmetry and publication bias, suggesting small studies overestimate the effect [[15](#_ENREF_15)]. | 11-12 |
| Additional analysis | 23 | | The sensitivity analysis included examination of estimates according to study size; the decade and regions in which studies were conducted; study quality as reviewed by the CCAT; use of adjusted or unadjusted estimates; dementia populations compared to non-dementia populations; different follow-up periods; and long term care in comparison to acute care utilisation; and the impact of different exposure measures. Summary estimates for each of the sensitivity analyses are displayed in Table 3. Given significant heterogeneity was found in the overall effect size, a meta-regression was conducted to investigate the contribution of different study characteristics to the level of heterogeneity.[[16](#_ENREF_16), [17](#_ENREF_17)] Results of the meta-regression are displayed in Tables 4 and 5. | 11-14 |
| **DISCUSSION** | | | |  |
| Summary of evidence | 24 | | Overall, the results suggest that, while carer stress has a significant effect on subsequent institutionalisation of care recipients, the size of this effect is negligible. This negligible effect size was reinforced in the sensitivity analysis. Whether the exposure was a measure of burden, stress, distress or depression the effect size remained very small to negligible. Similarly, whether the outcome was acute or long-term care the effect size was very small to negligible. This was also the case when examining estimates with particular measures such as the ZBI. Further, the effect size for studies with estimates that had adjusted for other factors was found to be substantially lower than with studies with un-adjusted estimates. While the un-adjusted estimate is larger than the adjusted, the un-adjusted estimate effect size is still small according to Cohen’s guidelines [[18](#_ENREF_18)]. Taken together, the findings suggest that other factors, such as the characteristics of the care recipient or the health system in which caring takes place, may be more crucial in institutional placement than carer stress.  These findings suggest that, over time, as studies have increased in size, quality has improved and more factors have been taken into account the size of the effect found has reduced. This would suggest that the significant association found between carer stress and institutionalisation in the initial studies in this area may have led to the belief that higher levels of carer stress could undermine the sustainability of homecare, however in later years this does not appear to have been critically evaluated. | 14-18 |
| Limitations | 25 | | Given the lack of clarity and consequential interchangeability in the measurement of psychological morbidity, the search strategy had to incorporate a wide number of exposures that are measured under the umbrella term ‘carer stress’. It could be argued that these exposures represent distinct concepts that when pooled may result in misclassification bias. However, there is theoretical and methodological support to pool the exposure measures. Firstly, Cramer et al. have developed a network approach to mental disorders and comorbidity where symptoms are viewed not as indicators of latent conditions but as components in a network.[[19](#_ENREF_19)] Employing this network model Borsboom et al. showed that half of the symptoms in the DSM-IV are connected.[[20](#_ENREF_20)] Secondly, to enable pooling the primary measure of effect was the standardized mean difference using a random effects model as recommended by Tak et al.[[11](#_ENREF_11)] By standardization the results were transformed to a common scale and the random effects model combined data under the assumption that the effect is not fixed between populations but varies around a typical value. | 18-19 |
| Conclusions | 26 | | The results of this review suggest that while carer stress has a significant effect on subsequent institutionalisation of care recipients, the size of this effect is negligible. The negligible effect size was reinforced in the sensitivity analysis, irrespective of the type of measure used. These results are at odds with the strong contention that higher levels of carer stress could undermine the sustainability of home care and suggest that publication bias or at least small study effects have contributed to this belief.  The findings should not be interpreted as undermining the significance of chronic stress on a carer. The level of carer stress experienced by the carer may be important both of itself and for its potential impact on a carer’s well-being and physical health. However, according to the present results, carer stress does not appear to be a crucial factor in institutionalisation of care recipients. Future research should look elsewhere for the determinants of institutionalisation in older care recipients. | 19-20 |
| **FUNDING** | | | |  |
| Funding | 27 | | This work was funded by the HRB in Ireland under Grant No. PHD/2007/16. | 20 |

# References

1. Deeken JF, Taylor KL, Mangan P, Yabroff KR, Ingham JM. Care for the caregivers: a review of self-report instruments developed to measure the burden, needs, and quality of life of informal caregivers. Journal of pain and symptom management. 2003;26(4):922-53.

2. Gaugler JE, Duval S, Anderson KA, Kane R. Predicting nursing home admission in the U.S: a meta-analysis. BMC Geriatrics. 2007;7(1):1-14.

3. Luppa M, Luck T, Weyerer S, Konig HH, Brahler E, Riedel-Heller SG. Prediction of institutionalization in the elderly. A systematic review. Age and ageing. 2010;39(1):31-8.

4. Miller EA, Weissert WG. Predicting Elderly People’s Risk for Nursing Home Placement, Hospitalization, Functional Impairment, and Mortality: A Synthesis. Medical Care Research and Review. 2000;57(3):259-97.

5. García-Pérez L, Linertová R, Lorenzo-Riera A, Vázquez-Díaz JR, Duque-González B, Sarría-Santamera A. Risk factors for hospital readmissions in elderly patients: a systematic review. QJM. 2011;104(8):639-51.

6. Campbell SE, Seymour DG, Primrose WR, project ftAp. A systematic literature review of factors affecting outcome in older medical patients admitted to hospital. Age and ageing. 2004;33(2):110-5.

7. Gaugler JE, Yu F, Krichbaum K, Wyman JF. Predictors of nursing home admission for persons with dementia. Medical care. 2009;47(2):191-8.

8. Crowe M, Sheppard L. A general critical appraisal tool: an evaluation of construct validity. International journal of nursing studies. 2011;48(12):1505-16.

9. Crowe M, Sheppard L, Campbell A. Reliability analysis for a proposed critical appraisal tool demonstrated value for diverse research designs. Journal of Clinical Epidemiology. 2012;65(4):375-83.

10. Crowe M, Sheppard L, Campbell A. Comparison of the effects of using the Crowe Critical Appraisal Tool versus informal appraisal in assessing health research: a randomised trial. International Journal of Evidence-Based Healthcare. 2011;9(4):444-9.

11. Tak LM, Meijer A, Manoharan A, de Jonge P, Rosmalen JG. More than the sum of its parts: meta-analysis and its potential to discover sources of heterogeneity in psychosomatic medicine. Psychosomatic medicine. 2010;72(3):253-65.

12. Kontopantelis E, Reeves D. METAEFF: Stata module to perform effect sizes calculations for meta-analyses 2011 [cited 2013 11th November 2013]. Available from: <http://EconPapers.repec.org/RePEc:boc:bocode:s457072>.

13. Higgins JPT, Thompson SG, Deeks JJ, Altman DG. Measuring inconsistency in meta-analyses. 2003;327(7414):557-60.

14. Thompson SG, Higgins J. How should meta‐regression analyses be undertaken and interpreted? Statistics in medicine. 2002;21(11):1559-73.

15. Steichen T. Tests for publication bias in meta-analysis. In: Sterne J, editor. Meta-analysis in Stata: An updated collection from the Stata Journal. Texas: Stata Press; 2009.

16. Sharp S. Meta-analysis regression. In: Sterne J, editor. Meta-analysis in Stata: An updated collection from the Stata Journal. Texas: Stata Press; 2009.

17. Harbord R, Higgins J. Meta-regression in Stata. In: Sterne J, editor. Meta-analysis in Stata: An updated collection from the Stata Journal. Texas: Stata Press; 2009.

18. Cohen J. Statistical power analysis for the behavioral sciences. 2nd ed. Hillsdale, NJ: Lawrence Earlbaum Associates; 1988.

19. Cramer AO, Waldorp LJ, van der Maas HL, Borsboom D. Comorbidity: a network perspective. The Behavioral and brain sciences. 2010;33(2-3):137-50; discussion 50-93.

20. Borsboom D, Cramer AOJ, Schmittmann VD, Epskamp S, Waldorp LJ. The Small World of Psychopathology. PLoS ONE. 2011;6(11):e27407.
